# Compartment-specific mutational landscape of clonal hematopoiesis

**DOI:** 10.1038/s41375-022-01700-3

**Published:** 2022-09-21

**Authors:** Luise Hartmann, Judith S. Hecker, Maja Rothenberg-Thurley, Jennifer Rivière, Madlen Jentzsch, Bianka Ksienzyk, Michèle C. Buck, Mark van der Garde, Luise Fischer, Susann Winter, Martina Rauner, Elena Tsourdi, Heike Weidner, Katja Sockel, Marie Schneider, Anne S. Kubasch, Martin Nolde, Dominikus Hausmann, Jörg Lützner, Szymon Goralski, Florian Bassermann, Karsten Spiekermann, Lorenz C. Hofbauer, Sebastian Schwind, Uwe Platzbecker, Katharina S. Götze, Klaus H. Metzeler

**Affiliations:** 1 Laboratory for Leukemia Diagnostics, Department of Medicine III, University Hospital (LMU), Munich, Germany; 2 German Cancer Consortium (DKTK), CHOICE Consortium, Partner Sites, Munich/Dresden, Germany; 3 German Cancer Research Center (DKFZ), Heidelberg, Germany; 4 Department of Medicine III, Technical University of Munich (TUM), Klinikum rechts der Isar, Munich, Germany; 5 Department of Hematology and Cell Therapy, University Hospital Leipzig (UHL), Leipzig, Germany; 6 Department of Medicine I, University Hospital Carl Gustav Carus, Technical University of Dresden (TUD), Dresden, Germany; 7 Center for Healthy Aging, Technical University of Dresden (TUD), Dresden, Germany; 8 Orthopedic Center Bogenhausen (OZB), Munich, Germany; 9 Department of Orthopedic Surgery, Technical University of Dresden (TUD), Dresden, Germany; 10 Department of Orthopedic Surgery, University Hospital Leipzig (UHL), Leipzig, Germany; 11 TranslaTUM, Center for Translational Cancer Research, Technical University of Munich (TUM), Munich, Germany

**Keywords:** Genetics research, Haematological cancer, Cancer genetics, Translational research

## Abstract

Clonal hematopoiesis (CH) is characterized by somatic mutations in blood cells of individuals without hematologic disease. While the mutational landscape of CH in peripheral blood (PB) has been well characterized, detailed analyses addressing its spatial and cellular distribution in the bone marrow (BM) compartment are sparse. We studied CH driver mutations in healthy individuals (*n* = 261) across different anatomical and cellular compartments. Variant allele frequencies were higher in BM than PB and positively correlated with the number of driver variants, yet remained stable during a median of 12 months of follow-up. In CH carriers undergoing simultaneous bilateral hip replacement, we detected *ASXL1*-mutant clones in one anatomical location but not the contralateral side, indicating intra-patient spatial heterogeneity. Analyses of lineage involvement in *ASXL1*-mutated CH showed enriched clonality in BM stem and myeloid progenitor cells, while lymphocytes were particularly involved in individuals carrying the c.1934dupG variant, indicating different *ASXL1* mutations may have distinct lineage distribution patterns. Patients with overt myeloid malignancies showed higher mutation numbers and allele frequencies and a shifting mutation landscape, notably characterized by increasing prevalence of *DNMT3A* codon R882 variants. Collectively, our data provide novel insights into the genetics, evolution, and spatial and lineage-specific BM involvement of CH.

## Introduction

Clonal hematopoiesis (CH), the expansion of hematopoietic clones driven by somatic mutations in *DNMT3A*, *TET2, ASXL1* and other genes, has been identified in elderly individuals through analyses of peripheral blood (PB) cells [1–4]. Since, by definition [5], persons with CH have no cytopenias or other evidence of hematologic cancer, studies evaluating CH in the bone marrow (BM) compartment and its various hematopoietic precursor cell fractions are rare, and many questions concerning the distribution of CH clones in different cellular and anatomical compartments remain unanswered [6, 7]. We recently reported the frequency of CH in a large cohort of individuals undergoing hip replacement surgery, and identified a high prevalence of somatic mutations of 50% [8]. We observed an increasing frequency of CH with age, as previously reported, and a previously unreported association with autoimmune diseases.

In this current study, we expanded this unique cohort, which gave us access to BM and PB samples from older individuals without hematologic disease as well as paired BM samples from individuals who underwent simultaneous bilateral hip replacement, to dissect and compare the mutation spectrum of CH in different cellular and anatomical compartments. Specifically, we sought to address the following questions: First, how do mutation spectrum and clone sizes of CH differ between different tissue compartments, i.e., PB and BM. Second, do CH clones evolve over a time period of 12–18 months? Third, is there spatial heterogeneity of BM involvement by CH at different anatomical sites? Fourth, which BM cell lineages are involved in CH with *ASXL1* mutations—the third-most common CH driver mutation which has not yet been characterized in detail in the marrow compartment? Overall, our data demonstrate that CH exhibits intra-individual heterogeneity, with variations detected between affected cell lineages, tissue compartments (PB vs. BM) as well as between anatomical localizations. Our data suggest that further in-depth investigations into spatial heterogeneity of CH and the process of clonal evolution to myeloid neoplasia are warranted. As consensus guidelines regarding the management of CH are still anticipated, our study provides information that is relevant for clinical diagnosis and monitoring, and adds to our understanding of temporal and spatial evolution of CH in various tissue compartments.

## Materials and methods

### Sample collection

BM from femoral heads (*n* = 177), baseline PB samples (*n* = 84), or both (*n* = 21) were collected from 261 individuals without known hematologic disease undergoing hip arthroplasty for osteoarthritis between 07/2017 and 02/2021 within the German Cancer Consortium (DKTK, consortium “CHOICE”), including 169 patients described in a prior report (Table 1) [8]. Participants were asked to provide follow-up PB samples at 6-month intervals. BM or PB samples from patients with myelodysplastic neoplasms (MDS) were obtained within the BoHemE registry at the universities of Dresden and Leipzig (TUD/UHL), and samples from patients with acute myeloid leukemia secondary to MDS (sAML) from the Laboratory for Leukemia Diagnostics, University of Munich (LMU) [9]. All participants provided written informed consent, and our study was approved by the respective ethics committees in accordance with the Declaration of Helsinki (approval numbers TUM 538/16, LMU 19-220, TUD EK 393092016 and UHL 137/19-lk).

**Table 1:** Characteristics of participants who underwent total hip arthroplasty.

Variable	THA cohort	CH (VAF ≥ 1%)	Non-CH	non-adjusted *p* (CH vs. non-CH)
*n* (%)	261	127 (48.7)	134 (51.3)	
Center				0.20
TUM, *n* (%)	177 (68)	91 (51)	86 (49)	
TUD/UHL, *n* (%)	84 (32)	36 (43)	48 (57)	
Age [years], median (range)	70 (18–86)	72 (53–86)	68 (18–83)	<0.001
Male sex, *n* (%)	93 (36)	39 (30)	54 (40)	0.11
Blood counts				
Leukocytes [10^9^/l], median (range)	6.7 (3.6–11.7)	6.8 (4.0–11.6)	6.7 (3.6–11.7)	0.37
Hemoglobin [g/dl], median (range)	13.4 (8.0–18.4)	12.7 (8.0–16.9)	13.7 (8.0–18.4)	0.003
MCV [fL], median (range)	90.0 (80.0–102.9)	92.0 (82.0–102.0)	89.0 (80.0–102.9)	0.021
Platelets [10^9^/l], median (range)	275 (110–563)	275 (110–563)	274 (144–490)	0.70

Clinical characteristics were compared between groups using the chi-square test for categorical variables and the Wilcoxon–Mann–Whitney test for continuous variables.

*CH* clonal hematopoiesis, *MCV* mean corpuscular volume, *TUM* Technical University Munich, *TUD* Technical University Dresden, *UHL* University Hospital Leipzig, *THA* total hip arthroplasty, *VAF* variant allele frequency.

### Detection of CH driver variants in PB and BM samples

Targeted sequencing of 68 genes recurrently mutated in hematologic malignancies was used to identify non-synonymous variants with a variant allele frequency (VAF) threshold of ≥1%, as described previously [8]. To validate next generation sequencing (NGS) results for selected variants, mutation-specific digital droplet PCR (ddPCR) assays were developed using a competitive probe-approach. This approach has been shown to achieve a typical sensitivity of ≥0.05% [10].

### Lineage distribution of *ASXL1* variants in sorted mature and progenitor hematopoietic cells

BM samples from individuals with *ASXL1*-mutated CH were flow-sorted into eight different mature and progenitor cell fractions (granulocytes; B lymphocytes; T lymphocytes; CD34^+^ precursors; hematopoietic stem cells (HSC); common myeloid progenitors (CMP); granulocyte-monocyte progenitors (GMP); and megakaryocyte-erythroid progenitors (MEP)) as described in the Supplemental Methods, Supplementary Table 1 and Supplementary Fig. 1 and as reported previously [11].

## Results

### Mutational landscape of CH in the blood and bone marrow compartments

**Fig. 1:**
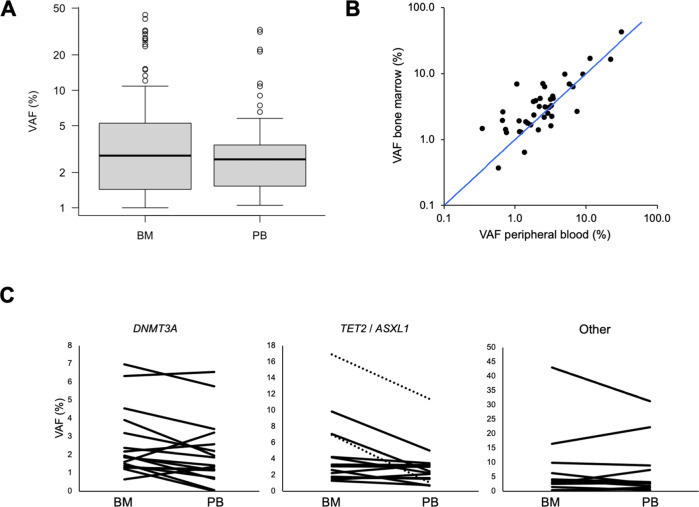
Comparison of variant allele frequencies (VAF) in CH among BM and PB samples. **A** VAF distribution in BM and PB samples. **B** Pairwise comparison of VAFs of 43 variants in paired BM and PB samples. The blue line represents equal VAFs in both compartments. **C** Pairwise comparison of VAFs in paired BM and PB samples, according to mutated gene. Left panel: *DNMT3A* variants (*n* = 17); middle panel: *TET2* variants (*n* = 12; solid lines) and *ASXL1* variants (*n* = 2; dashed lines); right panel, non-DTA variants (*n* = 12).

We report on a cohort of 261 individuals without known hematologic disease (median age, 70 years; range: 18–86) undergoing hip replacement surgery, including 169 subjects included in a previous report (Table 1) [8]. This gave us the unique opportunity to study CH driver variants not only in PB, but also in the BM compartment. In total, we identified 199 variants in 29 different genes, including 18 recurrently mutated drivers (Supplementary Table 2). CH drivers with VAFs of ≥1% or ≥2% (i.e., CH of indeterminate potential, CHIP [5]) were detected in 127 (49%) and 92 (35%) of 261 individuals, respectively. Allele frequencies of CH driver variants identified in BM (*n* = 144 variants in 92 individuals, median VAF, 2.8%, range, 1–44%) were similar to those found in PB (*n* = 55 in 35 individuals, median VAF, 2.6%, range, 1–33%; *p* = 0.6; Mann–Whitney *U*-test; Fig. 1A). In a subset of 21 individuals with CH and a total of 43 detected variants, we were able to analyze paired femoral head BM and PB samples obtained simultaneously (Fig. 1B, C and Supplementary Table 3). In this pairwise comparison, allele frequencies were higher in the BM compartment compared to the corresponding PB sample for 31 of 43 variants (72%, *p* = 0.0031; Wilcoxon signed-rank test). Forty-one of the 43 variants were detectable in both compartments. In three individuals, there was discordance between the mutation spectra observed in BM and PB (Supplementary Table 3). In one participant, 2 out of 3 variants present in the BM were also detectable in the PB, whereas one *DNMT3A* variant was present in the BM at a VAF of 1.2% (10/828 reads), yet undetected in the PB by NGS (0/874 reads). By mutation-specific ddPCR, this variant was detected in PB at a VAF of 0.08% (Supplementary Table 4). In another participant with a total of 5 variants, a *BCOR* mutation was found in the PB with a VAF of 1.2% (9/734 reads), but undetected in the corresponding BM sample (0/700 reads). Validation by ddPCR was technically unsuccessful for this variant. Finally, in a third patient, a *DNMT3A* variant was present at a VAF of 1.5% in the BM but undetectable by NGS from PB. This variant was identified at a VAF of 0.3% in PB by mutation-specific ddPCR. Overall, analyses of paired samples show that CH clones detectable in the marrow can also be identified in blood, although in most instances clone sizes tend to be slightly larger in BM than in matched PB samples. From a diagnostic standpoint, in all participants with CH who had paired samples analyzed, ≥1 variant was identified by NGS in the PB specimen, indicating that, when screening for CH, analyses of PB are adequate and analyses of BM specimens do not afford higher sensitivity.

**Fig. 2:**
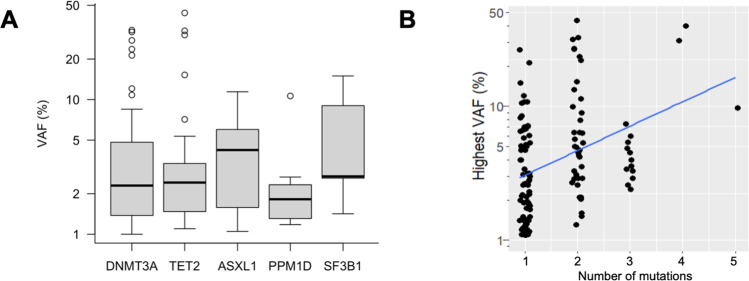
Variant allele frequencies (VAF) in CH. **A** VAF distributions for the five most frequently mutated genes. **B** Association between number of mutations and highest VAF per individual.

The median number of variants in individuals with CH was 1 (range, 1–5; 74/127 persons with CH [58%] carried a single variant). *DNMT3A*, *TET2*, and *ASXL1* were the most commonly affected genes with mutation frequencies of 28% (73/261 individuals), 17% (37/261) and 4% (11/261), respectively. VAF distributions of genes recurrently altered in ≥5 individuals (i.e., *DNMT3A*, *TET2*, *ASXL1*, *PPM1D* and *SF3B1*) did not differ significantly (*p* = 0.5, Kruskal–Wallis test; Fig. 2A). In persons with a higher total number of mutations, the largest detected VAF was significantly higher than in individuals with fewer mutations (*p* < 0.001, Spearman’s *ρ* = 0.37; Fig. 2B). In most persons with large-clone CHIP, defined as a largest VAF > 20%, the driver variant with the largest VAF was accompanied by one or more variants with lower allele frequencies, likely representing clonal heterogeneity in the form of subclonal variants and/or additional smaller clones (Supplementary Table 5). While such heterogeneity was more commonly detected in persons with large-clone CHIP, it remains unclear whether this finding reflects insufficient sensitivity of our assay to detect subclones within smaller clones, or whether clones with a stronger proliferative advantage and/or longer evolutionary history are more likely to acquire additional drivers.

### Evidence for spatial heterogeneity of CH in individuals undergoing simultaneous bilateral hip replacement surgery

**Fig. 3:**
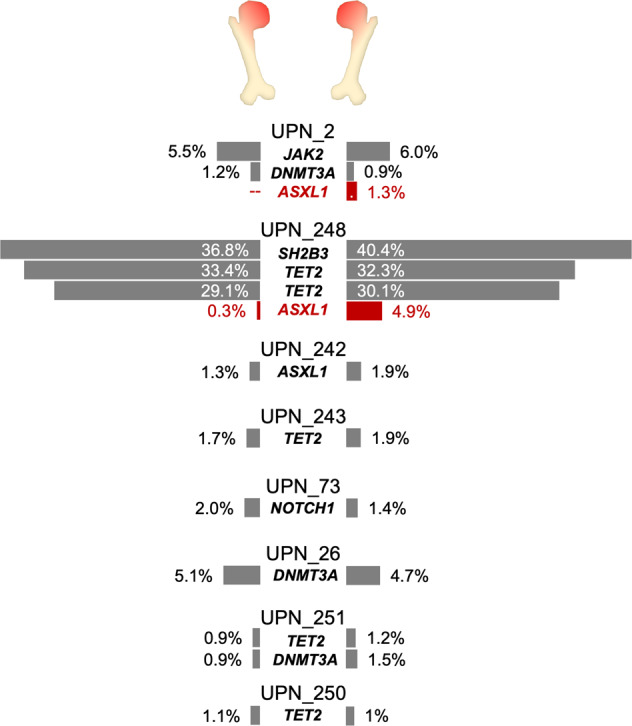
Spatial heterogeneity of clonal hematopoiesis in the bone marrow compartment as detected by NGS. Spatial heterogeneity was observed in two individuals (UPN_2 and UPN_248); mutations with significantly different VAFs (*p* < 0.01) are shown in red.

We analyzed 11 individuals who underwent bilateral simultaneous hip replacement (median age: 66 years, range: 58–75), providing us with the unique opportunity to study spatial heterogeneity of CH in the BM compartment. We identified CH in eight of these 11 persons (Fig. 3 and Supplementary Table 6). In six individuals, comparisons between the left and the right femoral heads showed concordance in the detected variants, with no significant differences in the observed VAFs. However, in two individuals we noticed spatial differences both affecting *ASXL1* mutations. In one subject, we observed an *ASXL1* nonsense variant at a VAF of 1.3% (29/2307 reads) in one femoral head sample. In the paired contralateral sample, the same variant was detected in only 2 of 4015 NGS reads (VAF, 0.05%), falling within the observed background noise in the area of interest. For orthogonal validation of this finding, we established a mutation-specific ddPCR assay, which confirmed a >20-fold difference in the relative size of the *ASXL1*-mutated clone between the two anatomical sites (Supplementary Table 4). In a second individual, we observed concordance between both femoral heads for two mutations in *TET2*, while a minor clone carrying an *ASXL1* frameshift mutation showed stronger involvement in one femoral head compared to the other (NGS VAF, 4.9% vs. 0.3%, *p* < 0.0001). Again, mutation-specific ddPCR confirmed a >15-fold larger VAF in one of the two simultaneously obtained BM samples. Our data thus demonstrate that CH clones show spatially heterogeneous involvement of BM from different anatomical localizations, suggesting initiation and expansion of CH clones or subclones may initially be a localized process, and raising the possibility of specific BM niches conducive to clonal expansion.

### Tracking allelic burden of *ASXL1* variants in sorted BM cell fractions reveals differences in lymphoid compartment involvement

**Fig. 4:**
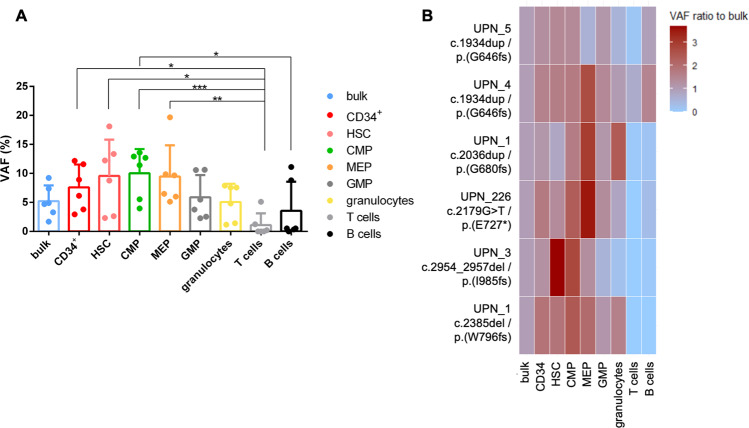
Lineage involvement of *ASXL1*-mutated CH. **A** Overall analysis of allelic burden according to the cell fraction of six *ASXL1* mutations in five CH individuals. Symbols: *p < 0.05, **p < 0.01, ***p < 0.001 using Friedman test followed by Nemenyi’s multiple comparison test. **B** Analysis of individual-specific allelic burden within each sorted cell fraction of the five *ASXL1*-mutated CH individuals.

Previous analyses of lineage involvement of CH clones have focused on *DNMT3A*- and *TET2*-mutated CH, and were mostly restricted to PB cells [6, 12]. Since we observed evidence of inter-compartment heterogeneity for *ASXL1* mutations (i.e., differences between BM and PB and between different BM sites), we studied involvement of various hematopoietic lineages and precursor cell stages by *ASXL1-*mutated CH. Analyzing sorted BM subpopulations from five individuals with CH and 6 distinct *ASXL1* mutations (Fig. 4A), we found that overall, *ASXL1* allelic burden in mature T-cells was significantly lower compared to progenitor cell fractions including CD34^+^ progenitors (*p* = 0.018), HSC (*p* = 0.012), CMP (*p* = 0.00041), and MEP (*p* = 0.0016). B-cells showed a significantly lower allelic burden when compared to CMP (*p* = 0.025, Friedman test followed by Nemenyi’s multiple comparison test).

When analyzing individual subjects, the *ASXL1* mutation was detected within the HSC fraction in all individuals (VAF > 1%; Supplementary Table 7). *ASXL1* mutation load was lower in T- and B-cells compared to bulk BM for 5/6 and 3/6 mutations, respectively (Fig. 4B and Supplementary Tables 7 and 8). In most individuals, *ASXL1* mutations were enriched in the HSC, CMP and/or MEP compartments relative to bulk BM cells. Of note, two individuals harbored the “hotspot” *ASXL1* variant c.1934dupG, which also had the highest VAFs of the 6 mutations (7.4% and 9.2%). Uniquely within those two individuals, the *ASXL1* variants were detected within the T-cell and B-cell fraction at VAFs >1% (5.1% and 11.1% in UPN_4; 1.2% and 8.8% in UPN_5, respectively), whereas allelic burden of all other *ASXL1* mutations was consistently <1% in T- and B-cells. While based on a limited number of patients, these data indicate that different *ASXL1* mutation types may show different lineage distribution patterns.

### Longitudinal analysis of CH dynamics show that clone sizes are stable during 6–24 months of follow-up

**Fig. 5:**
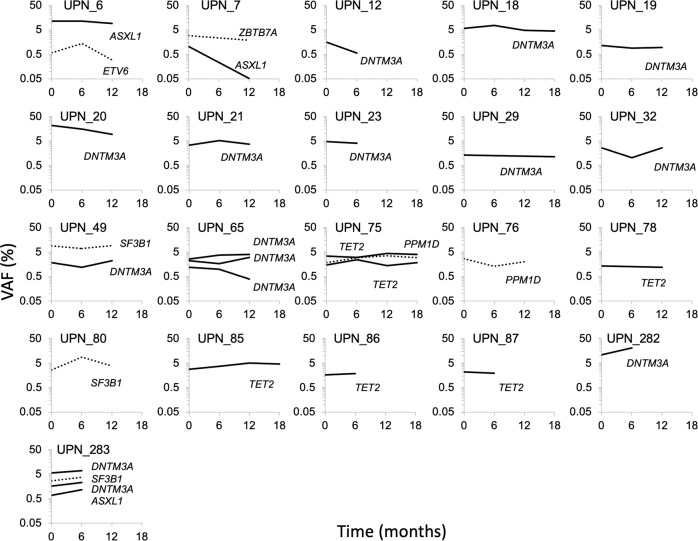
Longitudinal analyses of CH clonal evolution. A total of 31 variants in 21 individuals were analyzed in sequential PB samples for up to 18 months. Solid lines indicate DTA variants, dashed lines indicate non-DTA variants.

To study temporal evolution of CH clones, we sequentially analyzed PB samples from individuals without (*n* = 27) or with (*n* = 21) CH at baseline and during 6–24 months of follow-up (Fig. 5). Median duration of follow-up was 12 months for both cohorts. In 3/27 individuals without detectable CH at screening, four new CH driver mutations became detectable during follow-up and remained detectable in available subsequent samples (Supplementary Table 9). Importantly, manual re-analysis of sequencing data revealed that all four variants were already present in the respective screening samples, yet below the fixed detection threshold of our computational pipeline (i.e., 1% VAF).

In the cohort with detectable CH at baseline, we initially identified a total of 31 variants (Supplementary Table 10). Their median VAF was 2.6%, at screening, 2.9% at 6 months FU, and 3.0% at 12 months follow-up (*p* = 0.4 and *p* = 0.9, respectively, Wilcoxon signed-rank test). All but one variant were re-identified in available follow-up samples, albeit some dropped below 1% VAF and were only revealed by manual inspection of sequencing reads. Emergence of additional mutations was identified in two individuals, yet again, both variants were already present in the screening specimens at VAFs below the detection threshold (0.6% and 0.7%, respectively). Overall, our data demonstrate that while CH clone sizes slightly varied over time, no individual showed strong clonal expansion during 0.5–2 years of follow-up. Apparent “gains” or “losses” of CH variants usually affected variants with allele frequencies close to the limit of detection of the employed assay, and may most commonly represent random fluctuations in clone size or measurement variation, rather than true emergence or disappearance of clones.

### CH has a distinct driver mutation landscape compared to MDS and sAML, including a lower prevalence of *DNMT3A* codon R882 variants

**Fig. 6:**
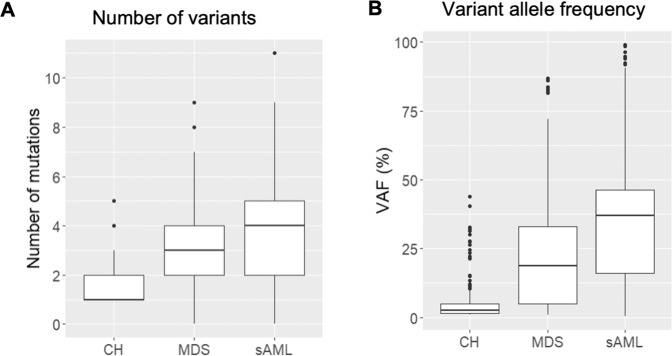
Driver mutation landscape in individuals with CH compared to patients with MDS or sAML. **A** Number of mutations per individual in CH, MDS and sAML. **B** Variant allele frequency distribution in CH, MDS and sAML.

CH is a potentially premalignant condition which may evolve into overt myeloid neoplasia, yet only a minority of individuals with CH undergo progression [13, 14]. To identify mutation patterns associated with malignant disease, regarding both the affected driver genes as well as specific mutations within recurrently mutated genes, we compared the genetic landscape of our CH cohort to patients with age-matched MDS (*n* = 91), or sAML (*n* = 123) (Supplementary Table 11), using a uniform assay for mutation analysis. Along the stages of this continuum of clonal alterations of myelopoiesis, there was an increase in the number of mutations per individual, from a median of 1 (range, 1–5) in CH to a median of 3 variants in MDS (range, 0–9; 7/91 MDS patients had no detectable variant) and a median of 4 variants in sAML (range, 0–11; 6/123 sAML patients had no detectable variant) (Fig. 6A and Supplementary Table 12). In parallel, the median VAF also increased across disease states (CH, 2.7%, range 1–44%; MDS, 19%, range 1–87%; and sAML, 37%, range 0.3–99%, Fig. 6B), indicating increasing clonality of the hematopoietic compartment and increasing genetic complexity with disease progression.

**Fig. 7:**
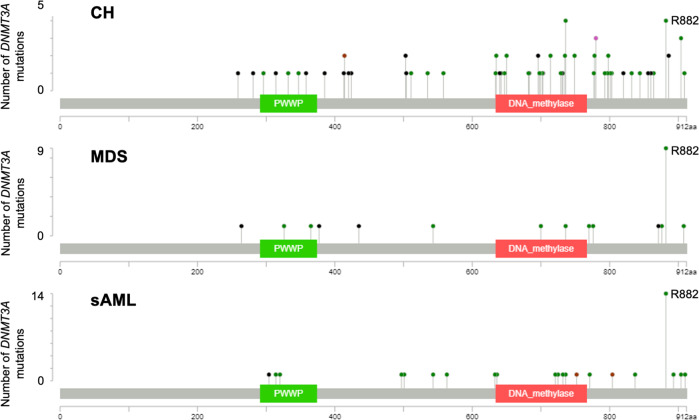
Intra-genic localization of *DNMT3A* variants in CH, MDS and sAML. Green dots indicate missense mutations, black dots indicate InDels, brown dots indicate nonsense mutations. Plots were generated using the MutationMapper tool by Gao et al. [24] and Cerami et al. [25].

While we observed an extensive overlap between genes affected by mutations across the three disease states, mutation patterns differed. The majority of individuals with CH harbored mutations in either *DNMT3A, TET2* or *ASXL1* (DTA) as previously published (Supplementary Fig. 2) [2, 3, 8]. Since DTA mutations occur across the spectrum of progression from CH to MDS and sAML, we studied the mutation spectrum within these genes to identify mutation characteristics associated with overt myeloid malignancy (Supplementary Table 13). Notably, in *DNMT3A*, variants at the p.R882 hotspot codon were less common in CH (4/87 *DNMT3A* mutations, 5%) compared to MDS (9/28, 32%) and sAML (14/37, 38%) (*p* < 0.001) (Fig. 7), suggesting that codon p.R882 mutations associate with disease progression and may have different functional implications compared to other variants in the gene. In contrast, in *TET2* and *ASXL1*, we did not observe differences in mutation location or the relative frequencies of missense, insertion/deletion (InDel), nonsense or splice site variants between CH, MDS and sAML (Supplementary Fig. 3).

Aside from DTA mutations, persons with CH also recurrently carried variants in other genes associated with myeloid neoplasms such as *JAK2*, *SRSF2* and *SF3B1* (Supplementary Fig. 2 and Supplementary Table 2). Overall, most variants found in CH affected epigenetic modifiers (53/199 variants [73%] in 102/127 individuals [83%]), while mutations in splicing factors, signaling pathways and transcription factors were more commonly associated with overt myeloid malignancy (Supplementary Fig. 2). Variants in *CBL, CEBPA, CSF3R, DDX41, ETNK1, ETV6, EZH2, FLT3, GATA2, IDH1, KIT, NPM1, NRAS, PTPN11* and *STAG2* were recurrently detected in MDS and sAML (Supplementary Table 11), but not in individuals with CH.

## Discussion

CH was initially described as an age-associated phenomenon that affects a substantial proportion of elderly individuals, most commonly beyond the age of 70 [2, 3, 8]. It quickly became clear that CH represents a potentially premalignant lesion with a propensity to progress to myeloid or lymphoid neoplasms. At the same time, CH also emerged as a novel risk factor for non-hematologic disorders, most prominently cardiovascular disease [15]. In the 5th edition of the WHO classification of haematolymphoid tumors, CH and CHIP have been recognized as potential premalignant ancestors of diverse myeloid neoplasms. However, due to the diversity of CH driver mutations, their clinical relevance for disease progression remains incompletely understood [5]. Recent studies using sensitive assays, allowing the detection of smaller clones, show that CH is present in most adults beyond the age of 60, and may become almost universal in the 8th and 9th decade of life [16, 17]. Consequently, it is evident that CH represents a heterogeneous spectrum of conditions with divergent health implications, and many instances of CH identified with sensitive methods may not carry strong clinical significance. In order to generate models for risk stratification that will allow us to assess the health implications of specific CH clones, a detailed characterization of the landscape of CH-associated genetic changes is needed.

In our study, we set out to perform an in-depth characterization of CH in various anatomical and cellular compartments, compare in detail the spectrum of CH driver variants to those found in manifest myeloid neoplasia, and study the stability and evolution of CH drivers in longitudinal samples obtained at multiple time points. Compared to other studies of CH which are often restricted to the analysis of PB leukocytes, and sometimes use assays with limited sensitivity such as whole-exome sequencing, we had access to BM samples and used a higher-sensitivity assay with a limit of detection of 1% VAF (corresponding to a clone size of 2% carrying a heterozygous somatic variant), allowing us to generate a detailed representation of the mutational spectrum and distribution in CH. One limitation of our study is the lack of single-cell analyses which would be needed to precisely recapitulate clonal hierarchy in individuals with multiple CH driver variants. Nonetheless, our study reveals several key insights. First, our use of a uniform assay platform to compare mutation spectra at different stages along the continuum from CH via MDS to sAML allows us to identify mutational signatures of progression. While all recurrent CH driver genes are also altered in established myeloid malignancies, epigenetic modifiers (in particular *DNMT3A* and *TET2*) are the most common lesions found in CH. Variants in *ASXL1*, while also prevalent in CH, are more common in MDS and AML. Variants in *IDH1* and *IDH2* are rare in CH and most common in sAML. While it has been shown that the oncometabolite 2-hydroxyglutarate, produced by the mutated IDH enzymes, inhibits *TET2* gene function [18], these differences in mutation frequencies suggest that *IDH1/IDH2* gene mutations may be functionally different, and associate with more advanced steps of myeloid carcinogenesis, than *TET2* variants. Likewise, mutations in signaling genes, splicing and transcription factors, tumor suppressors and the cohesin complex all are rare or absent in CH, and therefore likely associated with later steps of MDS/sAML development. Importantly, differences in the mutation landscape between CH and MDS/sAML may not only pertain to the genes affected, but also to the types and localization of variants found within a specific gene. Specifically, we show that *DNMT3A* mutations in CH are heterogeneous and spread across most parts of the gene’s coding sequence, whereas clustering at the well-known “hotspot” codon p.Arg882 is observed only in MDS and AML. These results again suggest that mutations affecting different regions of *DNMT3A* may have different functional implications, and that changes at codon 882 specifically associate with progression to myeloid neoplasia [19], as suggested by previous analyses. While our analyses reveal patterns of mutations associated with clonal evolution from CH to MDS and sAML, serial analyses of longitudinal samples did not reveal significant expansion of existing clones in CH over a 12- to 18-month period. This observation is in line with recently published data by Fabre et al. [20], demonstrating a clonal growth rate of ~5% per year. From a clinical viewpoint, our data indicate that frequent sequential testing (i.e., at intervals <12 months) is unlikely to reveal meaningful clonal evolution, and therefore unnecessary, in the majority of individuals with CH.

A second important aspect of our study is the heterogeneity observed between different cellular compartments. Overall, CH driver VAFs were relatively similar between PB and BM samples, indicating that use of PB samples for CH screening is adequate. Analyses of paired samples, however, showed that CH VAFs are generally higher in BM than PB. These differences may represent divergence in the cellular composition of both compartments, as well as in the degree of involvement of different lineages and maturation stages by the CH clone. We decided to study the latter aspect specifically for *ASXL1* mutations, as until now no data on lineage and differentiation- stage-specific involvement were available for this third-most common CH driver variant. Our data show preferential involvement of the HSC, CMP and MEP compartments relative to the GMP and more mature granulocyte compartment (CD15^+^), a finding that may contribute to higher VAFs in BM compared to PB samples. Similar to data reported for CH with *DNMT3A* and *TET2* driver mutations [6, 12], the B- and in particular the T-cell compartment showed the lowest VAFs, indicating that *ASXL1* mutations either occur in myeloid-primed progenitors or induce a myeloid differentiation bias themselves. The finding that the hotspot *ASXL1* variant c.1934dupG was detectable within the lymphoid cell compartments at a high VAF (while other *ASXL1* mutations were not), indicates that this mutation may affect particularly early multipotent progenitors in comparison to other *ASXL1* mutations or that the other *ASXL1* mutations are not compatible with lymphoid differentiation. Of note, the *ASXL1* c.1934 hotspot is located in a homopolymer sequence. While initial reports that the *ASXL1* c.1934dupG variant is not a somatic mutation [21] have been disproven [22, 23], this region is prone to false-positive sequencing findings. However, validation in large cohorts [9] and in the paired samples of this report show that our assay does not result in false-positive mutation calls at the VAFs reported here. Nevertheless, given the small sample number, our findings require further confirmation.

The availability of BM samples obtained simultaneously at two distinct anatomical sites (i.e., femoral heads obtained during bilateral hip replacement) gave us the unique opportunity to study for the first time not only lineage involvement, but also spatial heterogeneity of CH involvement of the BM compartment. Indeed, despite the limited number of paired bilateral femoral head samples, our data indicate that the distribution of CH clones is not always uniform throughout the BM. Using two independent methods, we demonstrate that in some patients with CH, the size of individual clones varies >10-fold between different anatomical locations. Our data thus indicate that CH can show spatially heterogeneous involvement of the marrow compartment, raising the question whether CH evolution might initially be restricted to certain anatomical locations. As CH arises from a single HSC at a specific location in the BM niche, the progress of expansion of the emerging clone to different regions of the BM niche will most probably require some time. Currently, very little is known about the dynamics and mechanisms of clonal migration and distribution. These processes may also be dependent on specific BM niches fostering clonal expansion. Overall, our findings warrant in-depth investigation of spatial distribution of clonal cell populations in the BM, and the relationship between mutated HSC and their anatomical BM niche. Collectively, our analyses contribute to a detailed understanding of the cellular compartments preferentially involved in CH and its distinctive properties compared to overt myeloid disease.

## Supplementary information

Supplemental Material

## Data Availability

The datasets generated during and/or analyzed during the current study are available from the corresponding author on reasonable request.
