# Supplementary material for: Compartment-specific mutational landscape of clonal hematopoiesis
Source: Leukemia. 2022 Sep 21;36(11):2647–55. doi: 10.1038/s41375-022-01700-3 (PMC9613457; doi:10.1038/s41375-022-01700-3)
Supplement: Supplementary file 1 — Supplemental Material [file 41375_2022_1700_MOESM1_ESM.pdf]

Supplemental material to Luise Hartmann, Judith S. Hecker, et al.:

Compartment-specific mutational landscape of clonal hematopoiesis

Supplemental Methods

Flow-sorting and genetic analyses of mature and precursor cell populations from BM samples

Antibodies used for cell sorting are listed in Supplemental Table 1.

After thawing BM samples, one fourth of the sample was stained with 100µl of an antibody mix for sorting mature cell populations (granulocytes, T lymphocytes, and B lymphocytes) along with $CD34^+$ cells (Supplemental Figure 1A). Dead cell exclusion was performed using DAPI 1µg/ml (BioLegend, #422801). The rest of the sample was sorted into fractions containing hematopoietic stem cells (HSC: $lin^-CD34^+CD38^-CD45RA^-CD90^+$), common-myeloid progenitors (CMP: $lin^-CD34^+CD38^+CD45RA^-CD123^+$), granulocyte-monocyte progenitors (GMP: $lin^-CD34^+CD38^+CD45RA^+CD123^+$) and megakaryocyte-erythroid progenitors (MEP: $lin^-CD34^+CD38^+CD45RA^-CD123^-$) (Supplemental Figure 1B). For this panel, dead cells were excluded using propidium iodide 0.4µg/ml (Sigma, #P4170). Sorting was performed using a BD FACS Aria III cell sorter and a 100µm nozzle.

DNA from flow-sorted cells (9.000-200.000 cells per population) was isolated using the QIAamp DNA Micro kit (Qiagen, Hilden, Germany). A custom unique molecular identifier-based QIAseq gene panel (Qiagen) targeting *ASXL1* exon 13 was used for library preparation, and sequencing was performed on a MiSeq instrument (Illumina, San Diego, USA). Variants were detected using the QIAGEN CLC Genomics Workbench. VAFs within flow-sorted cell populations were compared using the Friedman test followed by Dunn's multiple comparison test. *ASXL1* mutation VAFs in each flow-sorted population were compared to the same individual's bulk BM cells using Fisher's test, with multiple testing correction using the Benjamini-Hochberg procedure.

Supplemental Tables

Supplemental Table 1: List of antibodies used for FACS sorting of mature and progenitor BM cell populations.

Panel	Antibody	Conjugate	Dilution	Company	Catalogue Number
Mature	Anti-CD3	APC-Cy7	1:30	BioLegend	344818
Mature	Anti-CD15	FITC	1:10	Beckman Coulter	B36298
Mature	Anti-CD19	PE	1:30	BioLegend	302208
Mature	Anti-CD34	PE-Cy7	1:30	BioLegend	343616
Mature	Anti-CD45	APC	1:100	eBioscience	17-0459-42
Progenitor	Anti-CD4	biotin	1:100	BioLegend	300504
Progenitor	Anti-CD8a	biotin	1:100	BioLegend	301004
Progenitor	Anti-CD15	biotin	1:100	BioLegend	301914
Progenitor	Anti-CD19	biotin	1:100	BioLegend	302204
Progenitor	Anti-CD34	FITC	1:15	BD Pharmingen	555821
Progenitor	Anti-CD38	APC	1:25	BD Biosciences	345807
Progenitor	Anti-CD45	PerCP-Cy5.5	1:100	BioLegend	304028
Progenitor	Anti-CD45RA	PE-Cy7	1:20	BD Biosciences	560675
Progenitor	Anti-CD90	PE	1:25	BD Biosciences	555596
Progenitor	Anti-CD123	BV421	1:25	BioLegend	306018
Progenitor	Anti-CD235a	biotin	1:100	Invitrogen	13-9987-80
Progenitor	Straptavidin	APC-Cy7	1:100	BioLegend	405208

Supplemental Table 2: Variants identified in individuals with clonal hematopoiesis.

Subject	Gene	Variant	Mutation type	VAF [%]	Specimen
UPN_1	*ASXL1*	NM_015338.6:c.2036dup;p.(Gly680Argfs*38)	InDel	3.71	BM
UPN_1	*ASXL1*	NM_015338.6:c.2385del;p.(Trp796Glyfs*22)	InDel	5.93	BM
UPN_1	*STAT3*	NM_139276.2:c.1940A>T;p.(Asn647Ile)	Missense	1.79	BM
UPN_1	*TET2*	NM_001127208.2:c.5760A>T;p.(Lys1920Asn)	Missense	4.75	BM
UPN_1	*U2AF1*	NM_001025203.1:c.470A>C p.(Gln157Pro)	Missense	9.73	BM
UPN_10	*BCORL1*	NM_021946.4:c.4013G>A;p.(Arg1338Gln)	Missense	6.39	BM
UPN_10	*RAD21*	NM_006265.3:c.65A>G;p.(His22Arg)	Missense	4.49	BM
UPN_100	*ZBTB7A*	NM_015898.4:c.149C>T;p.(Ser50Leu)	Missense	3.18	BM
UPN_11	*BCORL1*	NM_021946.4:c.4853+1G>C;p.?	Splice site	10.86	BM
UPN_12	*DNMT3A*	NM_175629.2:c.2567_2568delAG;p.(Glu856Glyfs*7)	InDel	1.56	PB
UPN_12	*TET2*	NM_001127208.2:c.3954+5G>A;p.?	Splice site	1.13	PB
UPN_13	*DNMT3A*	NM_175629.2:c.2393T>C;p.(Leu798Pro)	Missense	1.00	BM
UPN_13	*TET2*	NM_001127208.2:c.5582G>A;p.(Gly1861Glu)	Missense	4.66	BM
UPN_14	*DNMT3A*	NM_175629.2:c.2339T>C;p.(Ile780Thr)	Missense	1.14	BM
UPN_14	*TET2*	NM_001127208.2:c.5686A>G;p.(Arg1896Gly)	Missense	5.34	BM
UPN_15	*DNMT3A*	NM_175629.2:c.1123-2A>G;p.?	Splice site	23.6	BM
UPN_15	*TET2*	NM_001127208.2:c.4079T>G;p.(Leu1360Arg)	Missense	1.19	BM
UPN_16	*DNMT3A*	NM_175629.2:c.2106T>G;p.(Asp702Glu)	Missense	3.59	BM
UPN_16	*IDH2*	NM_002168.3:c.678+1G>T;p.?	Splice site	1.07	BM
UPN_16	*TET2*	NM_001127208.2:c.1842dup;p.(Leu615Alafs*23)	InDel	1.18	BM
UPN_17	*DNMT3A*	NM_175629.2:c.1940T>G;p.(Leu647Arg)	Missense	4.47	BM
UPN_17	*TET2*	NM_001127208.2:c.4136C>A;p.(Ala1379Asp)	Missense	2.42	BM
UPN_17	*TET2*	NM_001127208.2:c.3116C>A;p.(Ser1039*)	Nonsense	2.81	BM
UPN_18	*DNMT3A*	NM_175629.2:c.2092T>G; p.(Trp698Gly)	Missense	5.77	PB
UPN_19	*DNMT3A*	NM_175629.2:c.2195T>C; p.(Phe732Ser)	Missense	1.15	PB
UPN_2	*ASXL1*	NM_015338.6:c.2083C>T;p.(Gln695*)	Nonsense	1.29	BM
UPN_2	*DNMT3A*	NM_175629.2:c.2141C>G;p.(Ser714Cys)	Missense	1.08	BM
UPN_2	*JAK2*	NM_004972.3:c.1849G>T, p.(Val617Phe)	Missense	5.96	BM
UPN_20	*DNMT3A*	NM_175629.2:c.2591T>A; p.(Met864Lys)	Missense	21.29	PB
UPN_201	*DNMT3A*	NM_175629.2:c.776delC;p.(Ala259Valfs*57)	InDel	1.53	BM
UPN_201	*DNMT3A*	NM_175629.2:c.942G>A;p.(Trp314*)	Nonsense	5.39	BM
UPN_201	*TET2*	NM_001127208.2:c.5618T>C;p.(Ile1873Thr)	Missense	2.78	BM
UPN_204	*TET2*	NM_001127208.2:c.3594+5G>T;p.?	Splice site	2.90	BM
UPN_204	*TET2*	NM_001127208.2:c.4585C>T;p.(Gln1529*)	Nonsense	2.44	BM
UPN_205	*DNMT3A*	NM_175629.2:c.1506del p.(Thr503Profs*148)	InDel	4.28	BM
UPN_205	*NFE2*	NM_001136023.3:c.814C>T;p.(Arg272*)	Nonsense	4.31	BM
UPN_206	*DNMT3A*	NM_175629.2:c.2408+6T>G;p.?	Splice site	1.41	BM
UPN_208	*DNMT3A*	NM_175629.2:c.2204A>G;p.(Tyr735Cys)	Missense	1.24	BM
UPN_21	*DNMT3A*	NM_175629.2:c.2047_2056del; p.(Tyr683Thrfs*19)	InDel	3.41	PB
UPN_213	*TET2*	NM_001127208.2:c.4791_4792del;p.(Phe1597Leufs*16)	InDel	1.39	BM
UPN_213	*TET2*	NM_001127208.2:c.444_447del;p.(Lys148Asnfs*3)	InDel	2.12	BM
UPN_214	*PPM1D*	NM_003620.4:c.1654C>T;p.(Arg552*)	Nonsense	10.63	BM

Subject	Gene	Variant	Mutation type	VAF [%]	Specimen
UPN_216	*PPM1D*	NM_003620.4:c.1654C>T;p.(Arg552*)	Nonsense	2.00	BM
UPN_218	*DNMT3A*	NM_175629.2:c.2086del;p.(Gln696Argfs*9)	InDel	1.16	BM
UPN_22	*DNMT3A*	NM_175629.2:c.2711C>T;p.(Pro904Leu)	Missense	6.64	BM
UPN_220	*DNMT3A*	NM_175629.2:c.2711C>T;p.(Pro904Leu)	Missense	1.16	BM
UPN_222	*TET2*	NM_001127208.2:c.2340_2343del;p.(Val781Lysfs*31)	InDel	1.77	BM
UPN_223	*DNMT3A*	NM_175629.2: c.2645G>A p.(Arg882His)	Missense	3.04	BM
UPN_225	*DNMT3A*	NM_175629.2:c.1674T>G;p.(Phe558Leu)	Missense	1.28	BM
UPN_225	*TET2*	NM_001127208.2:c.1441C>T;p.(Gln481*)	Nonsense	1.30	BM
UPN_226	*ASXL1*	NM_015338.6:c.2179G>T;p.(Glu727*)	Nonsense	2.67	BM
UPN_227	*PPM1D*	NM_003620.4:c.1709C>G;p.(Ser570*)	Nonsense	1.18	BM
UPN_23	*DNMT3A*	NM_175629.2:c.1506delT; p.(Thr503Profs*148)	InDel	4.73	PB
UPN_231	*DNMT3A*	NM_175629.2:c.2339T>C;p.(Ile780Thr)	Missense	1.64	BM
UPN_231	*DNMT3A*	NM_175629.2:c.2408G>A;p.(Arg803Lys)	Missense	1.79	BM
UPN_231	*DNMT3A*	NM_175629.2:c.842_855del;p.(Glu281Glyfs*38)	InDel	3.98	BM
UPN_232	*ASXL1*	NM_015338.6:c.1934dup;p.(Gly646Trpfs*12)	InDel	1.20	BM
UPN_232	*DNMT3A*	NM_175629.2:c.1603T>C;p.(Ser535Pro)	Missense	2.88	BM
UPN_232	*TET2*	NM_001127208.2:c.3765C>A;p.(Tyr1255*)	Nonsense	1.45	BM
UPN_233	*DNMT3A*	NM_175629.2:c.2207G>A;p.(Arg736His)	Missense	6.75	BM
UPN_235	*U2AF1*	NM_001025203.1:c.470A>G p.(Gln157Arg)	Missense	2.07	BM
UPN_235	*WT1*	NM_001198551.1:c.473G>A;p.(Arg158His)	Missense	2.86	BM
UPN_238	*DNMT3A*	NM_175629.2:c.1123-1G>C;p.?	Splice site	8.24	BM
UPN_24	*DNMT3A*	NM_175629.2:c.1258A>T;p.(Lys420*)	Nonsense	3.99	BM
UPN_242	*ASXL1*	NM_015338.6:c.2885_2886del;p.(Val962Alafs*7)	InDel	1.87	BM
UPN_243	*TET2*	NM_001127208.2:c.2919C>A;p.(Cys973*)	Nonsense	1.89	BM
UPN_245	*TP53*	NM_000546.5:c.623A>G;p.(Asp208Gly)	Missense	1.35	BM
UPN_248	*ASXL1*	NM_015338.6:c.2258_2259dup;p.(Thr754Profs*19)	InDel	4.87	BM
UPN_248	*SH2B3*	NM_005475.2:c.1522dup;p.(Arg508Profs*38)	InDel	40.42	BM
UPN_248	*TET2*	NM_001127208.2:c.5165del;p.(Pro1722Leufs*23)	InDel	30.10	BM
UPN_248	*TET2*	NM_001127208.2:c.5481_5482del;p.(Gln1828Alafs*17)	InDel	32.3	BM
UPN_25	*DNMT3A*	NM_175629.2:c.994G>A;p.(Gly332Arg)	Missense	2.30	BM
UPN_250	*TET2*	NM_001127208.2:c.2375C>A;p.(Ser792*)	Nonsense	1.10	BM
UPN_251	*TET2*	NM_001127208.2:c.5618T>C;p.(Ile1873Thr)	Missense	1.15	BM
UPN_251	*DNMT3A*	NM_175629.2:c.2530A>G;p.(Lys844Glu)	Missense	1.45	BM
UPN_252	*SF3B1*	NM_012433.3c.1986C>G;p.(His662Gln)	Missense	1.42	BM
UPN_252	*TET2*	NM_001127208.2:c.5686A>T;p.(Arg1896Trp)	Missense	2.10	BM
UPN_254	*SRSF2*	NM_003016.4:c.242A>T;p.(Asp81Val)	Missense	3.20	BM
UPN_254	*TET2*	NM_001127208.2:c.5650A>G;p.(Thr1884Ala)	Missense	43.88	BM
UPN_255	*PIGA*	NM_002641.3:c.217G>A;p.(Ala73Thr)	Missense	4.95	BM
UPN_26	*DNMT3A*	NM_175629.2:c.2332G>A;p.(Val778Met)	Missense	5.07	BM
UPN_266	*DNMT3A*	NM_175629.2:c.2207G>A;p.(Arg736His)	Missense	7.17	BM
UPN_267	*DNMT3A*	NM_175629.2:c.1238del;p.(Gly413Alafs*238)	InDel	1.00	BM
UPN_27	*DNMT3A*	NM_175629.2:c.1271del;p.(Pro424Hisfs*227)	InDel	1.49	BM
UPN_272	*SRP72*	NM_006947.3:c.869A>G;p.(Asn290Ser)	Missense	3.16	PB
UPN_275	*DNMT3A*	NM_175629.2:c.2657del;p.(Gln886Argfs*20)	InDel	1.94	PB

Subject	Gene	Variant	Mutation type	VAF [%]	Specimen
UPN_276	*DNMT3A*	NM_175629.2:c.1238dup;p.(Phe414Leufs*7)	InDel	1.42	PB
UPN_28	*DNMT3A*	NM_175629.2:c.2711C>T;p.(Pro904Leu)	Missense	5.33	BM
UPN_280	*PPM1D*	NM_003620.4:c.1332_1335del;p.(Phe445Glnfs*5)	InDel	1.24	PB
UPN_282	*DNMT3A*	NM_175629.2:c.2190_2215del;p.(Phe731*)	InDel	10.81	PB
UPN_283	*DNMT3A*	NM_175629.2:c.2207G>A;p.(Arg736His)	Missense	5.68	PB
UPN_283	*SF3B1*	NM_012433.3:c.1997A>G p.(Lys666Arg)	Missense	2.69	PB
UPN_288	*DNMT3A*	NM_175629.2:c.1903C>T;p.(Arg635Trp)	Missense	1.45	PB
UPN_288	*DNMT3A*	NM_175629.2:c.2086C>T;p.(Gln696*)	Nonsense	2.60	PB
UPN_29	*DNMT3A*	NM_175629.2:c.2322+2T>C;p.?	Splice site	1.35	PB
UPN_291	*DNMT3A*	NM_175629.2:c.2245C>T;p.(Arg749Cys)	Missense	1.64	PB
UPN_291	*DNMT3A*	NM_175629.2:c.1851+1G>A;p.?	Splice site	2.03	PB
UPN_3	*ASXL1*	NM_015338.6:c.2954_2957del;p.(Ile985Thrfs*7)	InDel	4.73	BM
UPN_30	*DNMT3A*	NM_175629.2:c.1948C>G;p.(Leu650Val)	Missense	1.06	PB
UPN_31	*DNMT3A*	NM_175629.2:c.1904G>A;p.(Arg635Gln)	Missense	6.54	PB
UPN_32	*DNMT3A*	NM_175629.2:c.2204A>G;p.(Tyr735Cys)	Missense	2.65	PB
UPN_33	*DNMT3A*	NM_175629.2:c.2330C>G;p.(Pro777Arg)	Missense	1.32	BM
UPN_34	*DNMT3A*	NM_175629.2:c.2393T>A, p.(Leu798His)	Missense	4.37	BM
UPN_35	*DNMT3A*	NM_175629.2:c.2401A>G; p.(Met801Val)	Missense	12.06	BM
UPN_36	*DNMT3A*	NM_175629.2:c.1015-2A>G;p.?	Splice site	4.76	BM
UPN_37	*DNMT3A*	NM_175629.2:c.1532G>A;p.(Gly511Glu)	Missense	1.49	BM
UPN_38	*DNMT3A*	NM_175629.2:c.2185C>T;p.(Arg729Trp)	Missense	8.48	BM
UPN_39	*DNMT3A*	NM_175629.2:c.2644C>T;p.(Arg882Cys)	Missense	1.06	BM
UPN_4	*ASXL1*	NM_015338.5:c.2512_2537dup; p.(Ser846Argfs*5)	InDel	11.42	PB
UPN_4	*ASXL1*	NM_015338.6:c.1934dup;p.(Gly646Trpfs*12)	InDel	6.07	BM
UPN_40	*DNMT3A*	NM_175629.2:c.1900A>T; p.(Ile634Phe)	Missense	1.07	BM
UPN_41	*DNMT3A*	NM_175629.2:c.1430-3C>G;p.?	Splice site	3.08	BM
UPN_42	*DNMT3A*	NM_175629.2:c.2377T>C;p.(Tyr793His)	Missense	1.74	BM
UPN_43	*DNMT3A*	NM_175629.2:c.2141C>G;p.(Ser714Cys)	Missense	2.14	BM
UPN_44	*DNMT3A*	NM_175629.2:c.1924G>A;p.(Gly642Arg)	Missense	1.17	BM
UPN_45	*DNMT3A*	NM_175629.2:c.2322+3A>G;p.?	Splice site	1.44	BM
UPN_49	*DNMT3A*	NM_175629.2:c.2727T>A; p.(Phe909Leu)	Missense	1.85	PB
UPN_49	*SF3B1*	NM_012433.3:c.1997A>C; p.(Lys666Thr)	Missense	9.01	PB
UPN_5	*ASXL1*	NM_015338.6:c.1934dup;p.(Gly646Trpfs*12)	InDel	7.01	BM
UPN_50	*DNMT3A*	NM_175629.2:c.2391C>A;p.(Asn797Lys)	Missense	1.51	BM
UPN_50	*NFE2*	NM_001136023.3:c.705dup;p.(Pro236Serfs*14)	InDel	3.09	BM
UPN_51	*DNMT3A*	NM_175629.2:c.2494A>G;p.(Thr832Ala)	Missense	6.42	BM
UPN_51	*ZRSR2*	NM_005089.3:c.370C>T;p.(Gln124*)	Nonsense	2.93	BM
UPN_52	*DNMT3A*	NM_175629.2:c.1949T>G, p.(Leu650Arg)	Missense	1.28	BM
UPN_52	*TP53*	NM_000546.5:c.536A>G, p.(His179Arg)	Missense	4.48	BM
UPN_53	*DNMT3A*	NM_175629.2: c.2644C>T, p.(Arg882Cys)	Missense	27.42	BM
UPN_53	*NFE2*	NM_001136023.3:c.578_581del, p.(Asn193Ilefs*12)	InDel	24.62	BM
UPN_54	*DNMT3A*	NM_175629.2:c.2332G>A;p.(Val778Met)	Missense	1.03	BM
UPN_54	*U2AF2*	NM_007279.2:c.977G>C;p.(Gly326Ala)	Missense	7.93	BM
UPN_55	*DNMT3A*	NM_175629.2:c.1668-2A>G;p.?	Splice site	2.32	BM

Subject	Gene	Variant	Mutation type	VAF [%]	Specimen
UPN_55	*MYD88*	NM_001172567.1:c.818T>C;p.(Leu273Pro)	Missense	27.86	BM
UPN_57	*DNMT3A*	M_175629.2:c.2096G>T; p.(Gly699Val)	Missense	1.44	BM
UPN_57	*DNMT3A*	NM_175629.2:c.1911_1914delGTCT;p.(Phe640Metfs*10)	InDel	3.33	BM
UPN_58	*DNMT3A*	NM_175629.2:c.2656C>T; p.(Gln886*)	Nonsense	1.20	BM
UPN_58	*DNMT3A*	NM_175629.2:c.1936+1G>T;p.?	Splice site	6.30	BM
UPN_59	*DNMT3A*	NM_175629.2:c.1071_1093del;p.(Thr358Profs*27)	InDel	5.38	BM
UPN_59	*DNMT3A*	NM_175629.2:c.2206C>T;p.(Arg736Cys)	Missense	31.70	BM
UPN_60	*DNMT3A*	NM_175629.2:c.2106_2107insA;p.(Leu703Thrfs*10)	InDel	1.23	BM
UPN_60	*DNMT3A*	NM_175629.2:c.1501_1508dup;p.(Leu504Metfs*150)	InDel	4.19	BM
UPN_61	*DNMT3A*	NM_175629.2:c.2045T>G;p.(Met682Arg)	Missense	2.39	BM
UPN_61	*DNMT3A*	NM_175629.2:c.2245C>T;p.(Arg749Cys)	Missense	3.29	BM
UPN_62	*DNMT3A*	NM_175629.2:c.2339T>C;p.(Ile780Thr)	Missense	1.22	PB
UPN_62	*DNMT3A*	NM_175629.2:c.1040T>C;p.(Leu347Pro)	Missense	32.70	PB
UPN_64	*DNMT3A*	NM_175629.2:c.1154del;p.(Pro385Argfs*22)	InDel	4.88	BM
UPN_64	*TP53*	NM_000546.5:c.524G>A;p.(Arg175His)	Missense	1.99	BM
UPN_64	*U2AF2*	NM_007279.2:c.866A>G;p.(Asn289Ser)	Missense	1.58	BM
UPN_65	*DNMT3A*	NM_175629.2:c.2580G>A;p.(Trp860*)	Nonsense	1.21	PB
UPN_65	*DNMT3A*	NM_175629.2:c.1851+1G>A;p.?	Splice site	2.57	PB
UPN_65	*DNMT3A*	NM_175629.2:c.2458G>T;p.(Glu820*)	Nonsense	2.20	PB
UPN_66	*DNMT3A*	NM_175629.2:c.2644C>T;p.(Arg882Cys)	Missense	1.96	PB
UPN_66	*DNMT3A*	NM_175629.2:c.1240_1245delTTCCAG; p.(Phe414_Gln415del)	InDel	1.18	PB
UPN_66	*IDH2*	NM_002168.3:c.419G>A;p.(Arg140Gln)	Missense	7.43	PB
UPN_69	*JAK2*	NM_004972.3:c.1849G>T p.(Val617Phe)	Missense	9.92	BM
UPN_69	*TET2*	NM_001127208.2:c.4045-2A>G;p.?	Splice site	3.56	BM
UPN_7	*ASXL1*	NM_015338.6:c.2644C>T; p.(Gln882*)	Nonsense	1.05	PB
UPN_7	*ZBTB7A*	NM_015898.4:c.1228T>A;p.(Tyr410Asn)	Missense	2.89	PB
UPN_70	*JAK2*	NM_004972.3:c.1849G>T p.(Val617Phe)	Missense	3.54	BM
UPN_70	*TET2*	NM_001127208.2:c.3789T>A;p.(Cys1263*)	Nonsense	2.60	BM
UPN_71	*KRAS*	NM_033360.3:c.35G>A;p.(Gly12Asp)	Missense	22.19	PB
UPN_71	*TET2*	NM_001127208.2:c.4393C>T;p.(Arg1465*)	Nonsense	3.02	PB
UPN_72	*MYD88*	NM_001172567.1:c.818T>C;p.(Leu273Pro)	Missense	2.38	BM
UPN_72	*TET2*	NM_001127208.2:c.2156del;p.(Leu719Cysfs*32)	InDel	1.21	BM
UPN_72	*TET2*	NM_001127208.2:c.5629A>G;p.(Lys1877Glu)	Missense	1.78	BM
UPN_72	*TET2*	NM_001127208.2:c.3647G>A;p. (Arg1216Gln)	Missense	2.42	BM
UPN_73	*NOTCH1*	ENST00000277541:c.7541_7542delCT;p.Pro2514fs	InDel	1.39	BM
UPN_74	*PHF6*	NM_032458.3:c.998A>T;p.(Asp333Val)	Missense	1.43	BM
UPN_75	*PPM1D*	NM_003620.3:c.1545_1546delGT; p.(Met515Ilefs*12)	InDel	1.82	PB
UPN_75	*TET2*	NM_001127208.2:c.3817T>C; p.(Cys1273Arg)	Missense	3.44	PB
UPN_75	*TET2*	NM_001127208.2:c.980C>G; p.(Ser327*)	Nonsense	1.50	PB
UPN_76	*PPM1D*	NM_003620.3:c.1636delC; p.(Leu546*)	InDel	2.66	PB
UPN_77	*PPM1D*	NM_003620.4:c.1573G>T; p.(Glu525*)	Nonsense	1.38	BM
UPN_78	*RUNX1*	NM_001754.4:c.664delT; p.(Ser222Profs*15)	InDel	2.69	PB
UPN_78	*TET2*	NM_001127208.2:c.1081C>T; p.(Gln361*)	Nonsense	1.37	PB

Subject	Gene	Variant	Mutation type	VAF [%]	Specimen
UPN_79	*RUNX1*	NM_001754.4:c.833C>A;p.(Pro278Gln)	Missense	1.76	BM
UPN_79	*TERT*	NM_198253.3:c.1573+6G>T;p.?	Splice site	4.96	BM
UPN_8	*BCOR*	NM_001123385.2:c.4163C>T; p.(Ala1388Val)	Missense	1.23	PB
UPN_8	*DNMT3A*	NM_175629.2:c.886G>A; p.(Val296Met)	Missense	3.21	PB
UPN_8	*TET2*	NM_001127208.2:c.4525A>T;p.(Lys1509*)	Nonsense	2.46	PB
UPN_8	*ZRSR2*	NM_005089.3:c.1338_1343dup;p.(Ser447_Arg448dup)	InDel	31.32	PB
UPN_80	*SF3B1*	NM_012433.3:c.2230G>C;p.(Ala744Pro)	Missense	2.61	PB
UPN_81	*SF3B1*	NM_012433.3:c.1873C>T, p.(Arg625Cys)	Missense	14.97	BM
UPN_82	*SRSF2*	NM_003016.4:c.283C>G;p.(Pro95Ala)	Missense	26.53	BM
UPN_85	*TET2*	NM_001127208.2:c.3885delC; p.(Tyr1295*)	InDel	2.81	PB
UPN_86	*TET2*	NM_001127208.2:c.5127dup;p.(Thr1710Tyrfs*3)	InDel	1.66	PB
UPN_87	*TET2*	NM_001127208.2:c.670G>T;p.(Glu224*)	Nonsense	2.16	PB
UPN_88	*TET2*	NM_001127208.2:c.456_457dup;p.(Ser153Phefs*3)	InDel	2.24	PB
UPN_89	*TET2*	NM_001127208.2:c.3954+5G>A;p.?	Splice site	2.10	BM
UPN_9	*BCOR*	NM_001123385.2:c.838G>A;p.(Val280Ile)	Missense	13.32	BM
UPN_9	*CXCR4*	NM_001008540.2:c.1045dup;p.(Glu349Glyfs*13)	InDel	1.92	BM
UPN_90	*TET2*	NM_001127208.2:c.4353del;p.(Arg1452Glufs*6)	InDel	5.17	BM
UPN_91	*TET2*	NM_001127208.2:c.5079_5082del;p.(Tyr1693*)	InDel	7.13	BM
UPN_92	*TET2*	NM_001127208.2:c.1486del;p.(Met496*)	InDel	1.30	BM
UPN_96	*TET2*	NM_001127208.2:c.3662G>A;p.(Cys1221Tyr)	Missense	3.24	PB
UPN_96	*TET2*	NM_001127208.2:c.3482G>C;p.(Arg1161Thr)	Missense	5.00	PB
UPN_97	*TET2*	NM_001127208.2:c.5650A>G;p.(Thr1884Ala)	Missense	1.11	BM
UPN_97	*TET2*	NM_001127208.2:c.4161C>A;p.(Asn1387Lys)	Missense	15.24	BM
UPN_98	*TET2*	NM_001127208.2:c.663_666delACAT;p.(His222Valfs*27)	InDel	2.14	PB
UPN_98	*TET2*	NM_001127208.2:c.1038_1039delAG;p.(Ala347Valfs*3)	InDel	3.27	PB
UPN_99	*U2AF2*	NM_007279.2:c.259_261delAAG;p.(Lys87del)	InDel	1.08	BM

Abbreviations: VAF, variant allele frequency; InDel, insertion/deletion variant; BM, bone marrow; PB, peripheral blood; UPN, unique patient number.

Supplemental Table 3: Comparison of variant allele frequencies in paired BM and PB samples from 21 individuals with clonal hematopoiesis.

			BM sample		PB sample	
**Subject**	**Gene**	**Variant**	**VAF [%]**	**Coverage**	**VAF [%]**	**Coverage**
UPN_6	*ASXL1*	c.2512_2537dup; p.(Ser846Argfs*5)	16.92	1876	11.42	1655
	ETV6	c.844dup;p.(Arg282Profs*18)	0.37	2685	0.58	1719
UPN_7	*ASXL1*	c.2644C>T; p.(Gln882*)	6.95	820	1.05	1045
	ZBTB7A	c.1228T>A;p.(Tyr410Asn)	2.50	1482	2.89	1383
UPN_8	***BCOR***	**c.4163C>T; p.(Ala1388Val)**	**ND**	**700**	**1.22**	**734**
	TET2	c.4525A>T;p.(Lys1509*)	7.06	1126	2.46	1342
	DNMT3A	c.886G>A; p.(Val296Met)	1.62	618	3.21	654
	DNMT3A	c.2339T>G;p.(Ile780Ser)	1.51	1323	0.06	3451
	ZRSR2	c.1338_1343dup;p.(Ser447_Arg448dup)	43.06	353	31.32	514
UPN_18	*DNMT3A*	c.2092T>G; p.(Trp698Gly)	6.97	717	5.77	762
UPN_19	*DNMT3A*	c.2195T>C; p.(Phe732Ser)	1.32	683	1.15	786
UPN_21	*DNMT3A*	c.2047_2056del; p.(Tyr683Thrfs*19)	4.55	857	3.41	586
UPN_29	*DNMT3A*	c.2322+2T>C;p.?	0.64	1713	1.35	1111
UPN_31	*DNMT3A*	c.1904G>A;p.(Arg635Gln)	6.33	1248	6.54	1469
UPN_49	*DNMT3A*	c.2727T>A; p.(Phe909Leu)	2.37	1054	1.85	758
	SF3B1	c.1997A>C; p.(Lys666Thr)	9.88	921	9.01	677
UPN_65	*DNMT3A*	c.2458G>T; p.(Glu820*)	3.18	1634	2.20	1407
	DNMT3A	c. 1851+1G>A; p.?	2.17	738	2.57	818
	DNMT3A	c.2580G>A;p.(Trp860*)	1.29	851	1.21	745
UPN_66	*DNMT3A*	c.1240_1245delTTCCAG;p.(Phe414_Gln415del)	1.91	1467	1.13	704
	DNMT3A	c.2644C>T;p.(Arg882Cys)	3.91	818	1.96	1124
	IDH2	c.419G>A;p.(Arg140Gln)	2.67	1422	7.43	1790
	DNMT3A	c.1429+1G>A;p.?	1.96	1018	0.66	1958
UPN_71	*TET2*	c.4393C>T;p.(Arg1465*)	3.07	489	3.02	364
	KRAS	c.35G>A;p.(Gly12Asp)	16.50	721	22.19	1005
UPN_75	*TET2*	c.980C>G; p.(Ser327*)	1.79	1119	1.50	1203
	TET2	c.3817T>C; p.(Cys1273Arg)	4.22	1207	3.44	1017
	PPM1D	c.1545_1546delGT; p.(Met515Ilefs*12)	3.75	773	1.82	881
UPN_76	*PPM1D*	c.1636delC; p.(Leu546*)	3.11	644	2.66	826
UPN_80	*SF3B1*	c.2230G>C;p.(Ala744Pro)	6.35	929	2.61	881
UPN_86	*TET2*	c.5127dup;p.(Thr1710Tyrfs*3)	1.66	1743	1.66	2046
UPN_88	*TET2*	c.456_457dup;p.(Ser153Phefs*3)	4.19	1337	2.24	848
UPN_96	*TET2*	c.3662G>A;p.(Cys1221Tyr)	3.29	3682	3.24	2408
	TET2	c.3482G>C;p.(Arg1161Thr)	9.84	2257	5.00	1341
	DNMT3A	c.1945G>T;p.(Val649Leu)	1.42	1615	0.73	2042
	TET2	c.822del;p.(Asn275Ilefs*18)	2.63	3226	0.67	4480
UPN_98	***DNMT3A****	**c.731del;p.(Pro244Leufs*72)**	**1.21**	**828**	**ND**	**874**
	TET2	c.663_666delACAT;p.(His222Valfs*27)	1.40	714	2.14	1167
	TET2	c.1038_1039delAG;p.(Ala347Valfs*3)	2.23	1165	3.27	1588
UPN_272	*SRP72*	c.869A>G;p.(Asn290Ser)	4.07	589	3.16	792
	TET2	c.3883T>G;p.(Tyr1295Asp)	1.28	1249	0.75	2259
UPN_276	*DNMT3A*	c.1238dup;p.(Phe414Leufs*7)	1.86	1557	1.42	1973
	STAG2	p.(Leu203Phe)	1.47	955	0.34	2336

* Highlighted variants were validated by digital droplet PCR, results are shown in Supplemental Table 4

Abbreviations: VAF, variant allele frequency; InDel, insertion/deletion variant; BM, bone marrow, PB, peripheral blood; ND, not detectable; UPN, unique patient number.

Supplemental Table 4: Orthogonal validation by mutation-specific digital droplet PCR of variants identified in paired BM and PB samples (UPN_98 and UPN_8) and in paired bone marrow samples from individuals undergoing simultaneous bilateral hip replacement (UPN_2 and UPN_248).

Subject	Gene	Variant	Sample	VAF [%]
UPN_98	*DNMT3A*	c.731del;<br>p.(Pro244Leufs*72)	BM	0.831
			PB	0.084
UPN_8	*DNMT3A*	c.2339T>G;<br>p.(Ile780Ser)	BM	1.584
			PB	0.267
UPN_2	*ASXL1*	c.2083C>T;<br>p.(Gln695*)	Hip 1	1.087
			Hip 2	0.050
UPN_248	*ASXL1*	c.2258_2259dup;<br>p.(Thr754Profs*19)	Hip 1	0.274
			Hip 2	4.154

Abbreviations: VAF, variant allele frequency; BM, bone marrow, PB, peripheral blood; UPN, unique patient number.

Supplemental Table 5: Overview of variants found in patients with 'large-clone' CHIP.

Large-clone CHIP was defined as at least one variant with a VAF >20% (highlighted in red in the table). Subjects are grouped according to their clonal profile (single variants, multiple variants with one "dominant" variant, or several variants with VAF >20%).

Mutational profile		UPN	Gene	Variant	Mutation type	VAF [%]
Single variant		UPN_82	*SRSF2*	NM_003016.4:c.283C>G;p.(Pro95Ala)	Missense	26.5
		UPN_20	*DNMT3A*	NM_175629.2:c.2591T>A; p.(Met864Lys)	Missense	21.3
Multiple variants	one dominant variant	UPN_254	*TET2*	NM_001127208.2:c.5650A>G;p.(Thr1884Ala)	Missense	43.9
			SRSF2	NM_003016.4:c.242A>T;p.(Asp81Val)	Missense	3.2
		UPN_62	*DNMT3A*	NM_175629.2:c.1040T>C;p.(Leu347Pro)	Missense	32.7
			DNMT3A	NM_175629.2:c.2339T>C;p.(Ile780Thr)	Missense	1.2
		UPN_59	*DNMT3A*	NM_175629.2:c.2206C>T;p.(Arg736Cys)	Missense	31.7
			DNMT3A	NM_175629.2:c.1071_1093del;p.(Thr358Profs*27)	InDel	5.4
		UPN_8	*ZRSR2*	NM_005089.3:c.1338_1343dup;p.(Ser447_Arg448dup)	InDel	31.2
			DNMT3A	NM_175629.2:c.886G>A; p.(Val296Met)	Missense	3.2
			TET2	NM_001127208.2:c.4525A>T;p.(Lys1509*)	Nonsense	2.5
			BCOR	NM_001123385.2:c.4163C>T; p.(Ala1388Val)	Missense	1.2
		UPN_55	*MYD88*	NM_001172567.1:c.818T>C;p.(Leu273Pro)	Missense	27.9
			DNMT3A	NM_175629.2:c.1668-2A>G;p.?	Splice site	2.3
		UPN_15	*DNMT3A*	NM_175629.2:c.1123-2A>G;p.?	Splice site	23.6
			TET2	NM_001127208.2:c.4079T>G;p.(Leu1360Arg)	Missense	1.2
		UPN_71	*KRAS*	NM_033360.3:c.35G>A;p.(Gly12Asp)	Missense	22.3
			TET2	NM_001127208.2:c.4393C>T;p.(Arg1465*)	Nonsense	3.0
	multiple variants with high allelic burden	UPN_248	*SH2B3*	NM_005475.2:c.1522dup;p.(Arg508Profs*38)	InDel	40.4
			TET2	NM_001127208.2:c.5481_5482del;p.(Gln1828Alafs*17)	InDel	32.3
			TET2	NM_001127208.2:c.5165del;p.(Pro1722Leufs*23)	InDel	30.1
			ASXL1	NM_015338.6:c.2258_2259dup;p.(Thr754Profs*19)	InDel	4.9
		UPN_53	*DNMT3A*	NM_175629.2: c.2644C>T, p.(Arg882Cys)	Missense	27.4
			NFE2	NM_001136023.3:c.578_581del, p.(Asn193Ilefs*12)	InDel	24.6

Abbreviations: VAF, variant allele frequency; InDel, insertion/deletion variant; UPN, unique patient number.

Supplemental Table 6: Paired-sample sequencing results for 11 patients undergoing bilateral hip replacements.

Hip pair	Variant	Hip 1		Hip 2		*p* (Fisher's exact test)
		Variant reads	VAF [%]	Variant reads	VAF [%]	
UPN_2	***ASXL1*:c.2083C>T;p.(Gln695*)**	**29/2307**	**1.29**	**2/4015**	**NA**	**<.0001**
	JAK2:c.1849G>T, p.(Val617Phe)	94/1577	5.96	141/2589	5.45	.49
	DNMT3A:c.2141C>G;p.(Ser714Cys)	20/2358	0.85	67/5779	1.15	.24
UPN_248	***ASXL1*:c.2258_2259dup;p.(Thr754Profs*19)**	**9/2779**	**0.32**	**144/2950**	**4.88**	**<.0001**
	TET2:c.5165del;p.(Pro1722Leufs*23)	432/1485	29.09	450/1495	30.1	.55
	TET2:c.5481_5482del;p.(Gln1828Alafs*17)	549/1644	33.39	575/1780	32.3	.51
	***SH2B3*:c.1522dup;p.(Arg508Profs*38)**	**565/1535**	**36.81**	**679/1680**	**40.42**	**.04**
UPN_242	*ASXL1*:c.2885_2886del;p.(Val962Alafs*7)	18/1402	1.28	40/2143	1.87	.22
UPN_243	*TET2*:c.2919C>A;p.(Cys973*)	26/1544	1.68	9/477	1.89	.84
UPN_73	*NOTCH1*:c.7541_7542delCT;p.Pro2514fs	11/552	1.99	7/503	1.39	.49
UPN_26	*DNMT3A*:c.2332G>A;p.(Val778Met)	76/1498	5.07	80/1688	4.74	.68
UPN_251	*TET2*:c.5618T>C;p.(Ile1873Thr)	25/2851	0.88	28/2252	1.15	.21
	DNMT3A:c.2530A>G;p.(Lys844Glu)	13/1447	0.90	20/1285	1.45	.16
UPN_250	*TET2*:c.2375C>A;p.(Ser792*)	23/1988	1.1	17/1785	0.95	.63
UPN_249	No variants identified					NA
UPN_240	No variants identified					NA
UPN_241	No variants identified					NA

Footnotes: Variants that were detected by manual inspection of sequencing reads are highlighted in red.

Abbreviations: VAF, variant allele frequency; NA, not applicable; UPN, unique patient number.

Supplemental Table 7: Allelic burden of eight flow-sorted subpopulations and bulk of five *ASXL1* mutated individuals.

		VAF in flow-sorted cell fractions								
**Subject**	***ASXL1* (NM_015338.6) variant**	**bulk**	**CD34$^+$**	**HSC**	**CMP**	**MEP**	**GMP**	**CD15$^+$**	**CD3$^+$**	**CD19$^+$**
UPN_226	c.2179G>T;p.(Glu727*)	1.70	2.94	2.33	3.97	6.00	2.29	1.42	0.00	0.53
UPN_1	c.2036dup;p.(Gly680Argfs*38)	3.31	3.81	2.64	5.56	9.86	3.79	7.91	0.23	0.54
	c.2385del;p.(Trp796Glyfs*22)	4.72	8.93	8.79	11.42	9.68	5.48	7.57	0.00	0.00
UPN_4	c.1934dup;p.(Gly646Trpfs*12)	7.38	11.60	12.23	12.70	19.67	10.60	7.58	5.12	11.11
UPN_5	c.1934dup;p.(Gly646Trpfs*12)	9.24	12.18	13.37	12.93	5.13	10.64	4.83	1.23	8.82
UPN_3	c.2954_2957del;p.(Ile985Thrfs*7)	4.90	6.15	18.11	13.58	6.48	2.56	1.17	0.00	0.34

Abbreviations: HSC, hematopoietic stem cells; CMP, common-myeloid progenitors; MEP, megakaryocyte-erythroid progenitors; GMP, granulocyte-monocyte progenitors; VAF, variant allele frequency; UPN, unique patient number.

Supplemental Table 8: Statistical analyses of differential clonal involvement of flow-sorted subpopulations and bulk marrow in *ASXL1*-mutated individuals.

Corrected *p*-values are indicated for the analysis of each pair of subpopulations using Fisher's test followed by correction for multiple hypothesis testing using the Benjamini-Hochberg procedure. Significant differences are indicated in red.

			HSC	CD34+	CMP	GMP	MEP	CD3+	CD19+	CD15+	bulk
UPN_226	NM_015338.6:c.2179G>T; p.(Glu727*)	HSC									
		CD34+	1.00								
		CMP	0.81	0.67							
		GMP	1.00	0.74	0.49						
		MEP	0.44	0.26	0.55	0.21					
		CD3+	0.052	0.00011	$6.83x10^{-5}$	0.0031	0.00011				
		CD19+	0.25	0.016	0.0032	0.099	0.0032	0.21			
		CD15+	0.74	0.27	0.10	0.55	0.052	0.017	0.29		
		bulk	0.74	0.27	0.099	0.73	0.052	0.0031	0.16	0.86	

			HSC	CD34+	CMP	GMP	MEP	CD3+	CD19+	CD15+	bulk
UPN_1	NM_015338.6:c.2036dup; p.(Gly680Argfs*38)	HSC									
		CD34+	0.61								
		CMP	0.15	0.15							
		GMP	0.61	1.00	0.22						
		MEP	0.0046	0.0016	0.057	0.0045					
		CD3+	0.0012	$3.24x10^{-10}$	$6.21x10^{-14}$	$1.59x10^{-8}$	$8.70x10^{-15}$				
		CD19+	0.017	$2.46x10^{-6}$	$3.17x10^{-9}$	$3.29x10^{-5}$	$4.07x10^{-11}$	0.36			
		CD15+	0.011	0.0058	0.18	0.013	0.55	$7.07x10^{-17}$	$3.77x10^{-12}$		
		bulk	0.86	0.68	0.051	0.71	0.00039	$3.17x10^{-9}$	$2.16x10^{-5}$	0.00084	

			HSC	CD34+	CMP	GMP	MEP	CD3+	CD19+	CD15+	bulk
UPN_4	NM_015338.6:c.1934dup; p.(Gly646Trpfs*12)	HSC									
		CD34+	0.92								
		CMP	0.97	0.78							
		GMP	0.64	0.78	0.46						
		MEP	0.016	0.0058	0.0058	0.0011					
		CD3+	0.0054	0.0058	0.00041	0.016	$1.16x10^{-9}$				
		CD19+	0.90	1.00	0.78	0.97	0.021	0.037			
		CD15+	0.15	0.19	0.065	0.35	$5.57x10^{-5}$	0.35	0.35		
		bulk	0.063	0.072	0.011	0.19	$7.08x10^{-8}$	0.29	0.22	1.00	

			HSC	CD34+	CMP	GMP	MEP	CD3+	CD19+	CD15+	bulk
UPN_5	NM_015338.6:c.1934dup; p.(Gly646Trpfs*12)	HSC									
		CD34+	0.74								
		CMP	0.91	0.80							
		GMP	0.67	0.75	0.71						
		MEP	0.0097	0.0046	0.0026	0.097					
		CD3+	$1.56x10^{-10}$	$1.15x10^{-15}$	$1.59x10^{-16}$	$2.5x10^{-6}$	0.0044				
		CD19+	0.097	0.088	0.038	0.67	0.13	$2.86x10^{-10}$			
		CD15+	0.0020	0.00041	$9.00x10^{-5}$	0.050	0.87	0.0026	0.041		
		bulk	0.15	0.13	0.077	0.74	0.088	$1.75x10^{-10}$	0.87	0.024	

			HSC	CD34+	CMP	GMP	MEP	CD3+	CD19+	CD15+	bulk
UPN_3	NM_015338.6:c.2954_2957del; p.(Ile985Thrfs*7)	HSC									
		CD34+	$2.64x10^{-7}$								
		CMP	0.054	0.0016							
		GMP	$3.45x10^{-15}$	0.031	$9.09x10^{-9}$						
		MEP	$1.82x10^{-6}$	0.87	0.0039	0.027					
		CD3+	$1.11x10^{-27}$	$7.83x10^{-8}$	$1.29x10^{-18}$	0.0010	$6.13x10^{-8}$				
		CD19+	$1.77x10^{-31}$	$1.79x10^{-7}$	$3.29x10^{-20}$	0.0068	$1.41x10^{-7}$	0.53			
		CD15+	$2.04x10^{-14}$	0.0034	$2.85x10^{-9}$	0.30	0.0028	0.061	0.20		
		bulk	$2.16x10^{-19}$	0.42	$2.32x10^{-8}$	0.060	0.33	$3.14x10^{-8}$	$3.98x10^{-8}$	0.0058	

			HSC	CD34+	CMP	GMP	MEP	CD3+	CD19+	CD15+	bulk
UPN_1	NM_015338.6:c.2385del; p.(Trp796Glyfs*22)	HSC									
		CD34+	1.00								
		CMP	0.74	0.57							
		GMP	0.49	0.31	0.048						
		MEP	1.00	0.90	0.90	0.38					
		CD3+	$2.00x10^{-6}$	$1.29x10^{-10}$	$1.32x10^{-12}$	$4.34x10^{-6}$	$8.39x10^{-6}$				
		CD19+	$9.69x10^{-6}$	$8.04x10^{-09}$	$1.24x10^{-10}$	$2.72x10^{-5}$	$2.72x10^{-5}$	1.00			
		CD15+	0.90	0.88	0.32	0.58	0.77	$1.37x10^{-7}$	$1.60x10^{-6}$		
		bulk	0.24	0.048	0.0030	0.88	0.24	$1.02x10^{-6}$	$1.55x10^{-5}$	0.32	

Abbreviations: HSC, hematopoietic stem cells; CMP, common-myeloid progenitors; MEP, megakaryocyte-erythroid progenitors; GMP, granulocyte-monocyte progenitors; VAF, variant allele frequency; UPN, unique patient number.

Supplemental Table 9: Longitudinal analysis in the baseline non-CH cohort over a time of up to 24 months.

Variants highlighted in red were not called during primary data analysis, but detected upon manual inspection of sequencing reads.

			VAF [%]				
**Subject**	**Gene**	**Variant**	**Screening**	**6 mo**	**12 mo**	**18 mo**	**24 mo**
UPN_281		none	none	none	NA	NA	NA
UPN_284		none	none	none	none	NA	NA
UPN_285		none	none	none	none	NA	NA
UPN_102	*DNMT3A*	NM_175629.2:c.[1257delT]; p.(Lys420Argfs*231)	0.70	NA	1.32	NA	2.00
UPN_103		none	none	NA	none	none	NA
UPN_104		none	none	NA	none	NA	NA
UPN_105		none	none	NA	none	NA	NA
UPN_106	*PRPF8*	NM_006445.4:c.3361A>G; p.(Asn1121Asp)	0.91	0.82	1.02	1.20	NA
UPN_107		none	none	NA	none	NA	NA
UPN_108		none	none	NA	none	NA	NA
UPN_109		none	none	NA	none	NA	NA
UPN_110		none	none	none	NA	NA	NA
UPN_111		none	none	none	NA	NA	NA
UPN_112		none	none	NA	none	NA	NA
UPN_113		none	none	none	none	NA	NA
UPN_114		none	none	NA	none	NA	NA
UPN_115		none	none	none	NA	NA	NA
UPN_116		none	none	none	none	NA	NA
UPN_117		none	none	none	none	NA	NA
UPN_118		none	none	none	NA	NA	NA
UPN_119		none	none	none	NA	NA	NA
UPN_120	*ASXL1*	NM_015338.6:c.2036dup; p.(Gly680Argfs*38)	1.16	1.78	NA	NA	NA
	TET2	NM_001127208.2:c.3986T>C; p.(Leu1329Pro)	1.05	1.42	NA	NA	NA
UPN_121		none	none	none	NA	NA	NA
UPN_122		none	none	none	none	NA	NA
UPN_124		none	none	NA	none	NA	NA
UPN_126		none	none	none	NA	NA	NA
UPN_127		none	none	NA	none	NA	NA

Abbreviations: VAF, variant allele frequency; mo, months; NA, not analyzed; UPN, unique patient number.

Supplemental Table 10: Longitudinal analysis of CH temporal evolution over a time of up to 18 months.

Bold font indicates variants that emerged or dropped below the limit of detection during follow-up. Variants in red were not called during primary data analysis, but detected upon manual inspection of sequencing reads.

			VAF [%]			
**Subject**	**Gene**	**Variant**	**Screening**	**6 mo**	**12 mo**	**18 mo**
UPN_49	*DNMT3A*	c.2727T>A; p.(Phe909Leu)	1.85	1.19	2.20	NA
	SF3B1	c.1997A>C; p.(Lys666Thr)	9.01	6.97	9.25	NA
UPN_85	*TET2*	c.3885delC; p.(Tyr1295*)	2.81	3.71	5.05	4.53
UPN_18	*DNMT3A*	c.2092T>G; p.(Trp698Gly)	5.77	7.57	4.75	4.54
UPN_75	*TET2*	c.980C>G; p.(Ser327*)	1.50	2.42	1.41	1.85
	TET2	c.3817T>C; p.(Cys1273Arg)	3.44	3.05	4.33	3.95
	PPM1D	c.1545_1546delGT; p.(Met515Ilefs*12)	1.82	2.93	3.56	2.9
UPN_65	*DNMT3A*	c.2458G>T; p.(Glu820*)	2.20	1.70	2.99	NA
	DNMT3A	c.1851+1G>A;p.?	2.57	3.70	3.99	NA
	DNMT3A	c.2580G>A;p.(Trp860*)	1.21	1.00	0.39	NA
UPN_6	*ASXL1*	c.2512_2537dup; p.(Ser846Argfs*5)	11.42	11.320	9.19	NA
	ETV6	**c.844dup;p.(Arg282Profs*18)**	**0.58**	**1.40**	**0.30**	**NA**
UPN_19	*DNMT3A*	c.2195T>C; p.(Phe732Ser)	1.15	0.89	0.95	NA
UPN_20	*DNMT3A*	c.2591T>A; p.(Met864Lys)	21.29	15.28	9.38	NA
UPN_21	*DNMT3A*	c.2047_2056del; p.(Tyr683Thrfs*19)	3.41	5.23	3.68	NA
UPN_76	*PPM1D*	c.1636delC; p.(Leu546*)	2.66	1.31	2.04	NA
UPN_23	*DNMT3A*	c.1506delT; p.(Thr503Profs*148)	4.73	4.07	NA	NA
UPN_80	*SF3B1*	c.2230G>C;p.(Ala744Pro)	2.61	8.73	3.80	NA
UPN_78	*TET2*	c.1081C>T; p.(Gln361*)	1.37	NA	1.20	NA
UPN_32	*DNMT3A*	c.2204A>G;p.(Tyr735Cys)	2.60	1.04	2.64	NA
UPN_12	*DNMT3A*	c.2567_2568delAG;p.(Glu856Glyfs*7)	1.56	0.56	NA	NA
UPN_87	*TET2*	c.670G>T;p.(Glu224*)	2.16	1.92	NA	NA
UPN_86	*TET2*	c.5127dup;p.(Thr1710Tyrfs*3)	1.66	1.89	NA	NA
UPN_29	*DNMT3A*	c.2322+2T>C;p.?	1.35	NA	NA	1.13
UPN_282	*DNMT3A*	c.2190_2215del;p.(Phe731*)	10.81	20.92	NA	NA
UPN_283	*DNMT3A*	c.890G>C;p.(Trp297Ser)	1.65	2.31	NA	NA
	DNMT3A	c.2207G>A;p.(Arg736His)	5.68	6.87	NA	NA
	SF3B1	c.1997A>G;p.(Lys666Arg)	2.69	3.70	NA	NA
	ASXL1	**c.1934dup p.(Gly646Trpfs*12)**	**0.68**	**1.16**	**NA**	**NA**
UPN_7	***ASXL1***	**c.2644C>T; p.(Gln882*)**	**1.05**	**NA**	**ND**	**NA**
	ZBTB7A	c.1228T>A;p.(Tyr410Asn)	2.89	NA	1.88	NA

Abbreviations: VAF, variant allele frequency; ND, not detected; mo, months; NA, not analyzed; UPN, unique patient number

Supplemental Table 11: Variants identified in patients with MDS or sAML.

Subject	Gene	Variant	Mutation type	VAF [%]
MDS_1	*CBL*	NM_005188.3:c.1244G>A:p.(Gly415Asp)	Missense	38.0
MDS_1	*TET2*	NM_001127208.2:c.3404G>A;p.(Cys1135Tyr)	Missense	36.8
MDS_1	*TP53*	NM_000546.5:c.827C>G;p.(Ala276Gly)	Missense	25.1
MDS_2	*DDX41*	NM_016222:c.1574G>A;p.R525H	Missense	4.3
MDS_2	*DNMT3A*	NM_175629.2: c.2645G>A p.(Arg882His)	Missense	5.1
MDS_2	*SRSF2*	NM_003016.4:c.284C>A p.(Pro95His)	Missense	4.7
MDS_3	*BCOR*	NM_001123385.2:c.3649del;p.(Arg1217Aspfs*21)	InDel	32.3
MDS_3	*BCORL1*	NM_021946.4:c.4230dup;p.(Glu1411*)	InDel	4.9
MDS_3	*DNMT3A*	NM_175629.2:c.2644C>A;p.(Arg882Ser)	Missense	19.3
MDS_3	*NRAS*	NM_002524.5:c.38G>T;p.(Gly13Val)	Missense	14.3
MDS_3	*U2AF1*	NM_001025203.1:c.101C>T;p.(Ser34Phe)	Missense	20.3
MDS_4	*RAD21*	NM_006265.3:c.742C>G;p.(Leu248Val)	Missense	37.1
MDS_4	*SF3B1*	NM_012433.3:c.2098A>G p.(Lys700Glu)	Missense	42.6
MDS_4	*TET2*	NM_001127208.2:c.3854_3856del;p.(Phe1285del)	InDel	30.5
MDS_4	*TET2*	NM_001127208.2:c.3197_3198dup;p.(Arg1067Leufs*16)	InDel	34.0
MDS_4	*TP53*	NM_000546.5:c.994-3C>G;p.?	Splice site	1.1
MDS_5	*BCOR*	NM_001123385.2:c.2340dup;p.(Thr781Hisfs*36)	InDel	9.5
MDS_5	*ETNK1*	NM_018638.4:c.731A>G;p.(Asn244Ser)	Missense	5.5
MDS_5	*RUNX1*	NM_001754.4:c.431T>C;p.(Leu144Pro)	Missense	18.8
MDS_5	*SRSF2*	NM_003016.4:c.284C>G p.(Pro95Arg)	Missense	25.8
MDS_5	*TET2*	NM_001127208.2:c.3908G>A;p.(Ser1303Asn)	Missense	16.1
MDS_5	*TET2*	NM_001127208.2:c.1630C>T;p.(Arg544*)	Nonsense	27.0
MDS_6	*IDH1*	NM_005896.3: c.394C>T p.(Arg132Cys)	Missense	4.3
MDS_7	*SRSF2*	NM_003016.4:c.284C>A p.(Pro95His)	Missense	11.6
MDS_7	*TET2*	NM_001127208.2:c.2147C>G;p.(Ser716*)	Nonsense	10.3
MDS_7	*RUNX1*	NM_001754.4:602G>A p.(Glu138Lys)	Missense	11.9
MDS_7	*STAG2*	NM_001042750.2:c.1644dup;p.(Thr549Tyrfs*11)	InDel	20.6
MDS_7	*BCOR*	NM_001123385.2:c.2972_2976del;p.(Leu991Profs*25)	InDel	21.9
MDS_8	*BCORL1*	NM_021946.4:c.1202C>T;p.(Thr401Met)	Missense	19.5
MDS_8	*GATA2*	NM_001145661.2:c.952G>A:p.(Ala318Thr)	Missense	17.3
MDS_8	*RUNX1*	NM_001754.4:c.749delinsCC;p.(Arg250Profs*11)	InDel	15.7
MDS_8	*SF3B1*	NM_012433.3:c.2098A>G p.(Lys700Glu)	Missense	18.0
MDS_9	*SF3B1*	NM_012433.3:c.2098A>G p.(Lys700Glu)	Missense	52.5
MDS_9	*TET2*	NM_001127208.2:c.4636C>T;p.(Gln1546*)	Nonsense	43.7
MDS_10	*TET2*	NM_001127208.2:c.4210C>T;p.(Arg1404*)	Nonsense	2.9
MDS_10	*TET2*	NM_001127208.2:c.3781C>T;p.R1261C	Missense	20.3
MDS_10	*ZRSR2*	NM_005089.3:c.900_901del;p.(Glu300Aspfs*16)	InDel	39.0
MDS_11	*ASXL1*	NM_015338.6:c.2904dup;p.(Asp969*)	InDel	20.6
MDS_11	*TET2*	NM_001127208.2:c.4183-2A>G;p.?	Splice site	2.4
MDS_11	*TET2*	NM_001127208.2:c.3532del;p.E1178Kfs*48	InDel	17.6
MDS_11	*TET2*	NM_001127208.2:c.4546C>T;p.(Arg1516*)	Nonsense	22.4
MDS_11	*TP53*	NM_000546.5:c.665C>T;p.(Pro222Leu)	Missense	40.2
MDS_11	*U2AF1*	NM_001025203.1:c.470A>C p.(Gln157Pro)	Missense	20.2

Subject	Gene	Variant	Mutation type	VAF [%]
MDS_12	*ASXL1*	NM_015338.6:c.1900_1922del;p.(Glu635Argfs*15)	InDel	2.1
MDS_12	*ASXL1*	NM_015338.6:c.2082_2092del;p.(Gln695Alafs*19)	InDel	15.3
MDS_12	*ETNK1*	NM_018638.4:c.731A>G;p.(Asn244Ser)	Missense	5.3
MDS_12	*U2AF1*	NM_001025203.1:c.470A>C p.(Gln157Pro)	Missense	20.2
MDS_13	*SETBP1*	NM_015559.3:c.2602G>A;p.Asp868Asn	Missense	4.7
MDS_13	*SETBP1*	NM_015559.3:c.2608G>A (p.Gly870Ser)	Missense	21.9
MDS_13	*SF3B1*	NM_012433.3:c.2098A>G p.(Lys700Glu)	Missense	26.4
MDS_13	*TET2*	NM_001127208.2:c.4956del;p.(Gln1652Hisfs*43)	InDel	1.1
MDS_13	*TET2*	NM_001127208.2:c.4097G>A;R1366H	Missense	1.8
MDS_13	*TET2*	NM_001127208.2:c.5071del;p.(Ser1691Leufs*4)	InDel	7.7
MDS_14	*SRSF2*	NM_003016.4:c.284C>T p.(Pro95Leu)	Missense	13.8
MDS_14	*TET2*	NM_001127208.2:c.1776T>G;p.(Tyr592*)	Nonsense	22.6
MDS_14	*TET2*	NM_001127208.2:c.2193del;p.(Gln731Hisfs*20)	InDel	24.8
MDS_14	*RUNX1*	NM_001754.4:c.1274C>T;p.(Pro425Leu)	Missense	14.2
MDS_15	*TET2*	NM_001127208.2:c.4108G>A;p.(Gly1370Arg)	Missense	3.8
MDS_15	*DNMT3A*	NM_175629.2:c.1131del;p.(Ser377Argfs*30)	InDel	1.6
MDS_15	*DNMT3A*	NM_175629.2:c.1628G>C;p.(Gly543Ala)	Missense	6.0
MDS_15	*TP53*	NM_000546.5:c.377A>G;p.(Tyr126Cys)	Missense	3.7
MDS_15	*KRAS*	NM_033360.4:c.35G>C (p.Gly12Ala)	Missense	1.1
MDS_16	*SRSF2*	NM_003016.4:c.284C>T p.(Pro95Leu)	Missense	17.2
MDS_16	*RUNX1*	NM_001754.4:c.422C>T;p.(Ser141Leu)	Missense	1.9
MDS_16	*RUNX1*	NM_001754.4:c.412G>T;p.(Glu138*)	Nonsense	23.6
MDS_17	*MPL*	NM_005373.2:c.1771T>G;p.(Tyr591Asp)	Missense	41.0
MDS_18	*DNMT3A*	NM_175629.2:c.2645G>C; p.(Arg882Pro)	Missense	28.0
MDS_18	*TET2*	NM_001127208.2:c.2553del; p.(Glu852Asnfs*21)	InDel	30.0
MDS_18	*TET2*	NM_001127208.2:c.2243del; p.(Leu748Tyrfs*3)	InDel	20.0
MDS_18	*TET2*	NM_001127208.2:c.3784C>T; p.(Arg1262Trp)	Missense	7.6
MDS_18	*SRSF2*	NM_003016.4:c.284C>T p.(Pro95Leu)	Missense	1.5
MDS_19	*ASXL1*	NM_015338.5:c.1900_1922del; p.(Glu635Argfs*15)	InDel	35.0
MDS_19	*EZH2*	NM_004456.4:c.1981A>T; p.(Lys661*)	Nonsense	45.0
MDS_19	*RUNX1*	NM_001754.4:c.492_493insGG; p.(Arg166Valfs*11)	InDel	13.0
MDS_19	*STAG2*	NM_001042749.2:c.1117-1G>C;p.?	Splice site	9.0
MDS_19	*TET2*	NM_001127208.2:c.4075C>T; p.(Arg1359Cys)	Missense	12.0
MDS_19	*TET2*	NM_001127208.2:c.1326delC; p.(Asn442Lysfs*5)	InDel	2.0
MDS_20	*ASXL1*	NM_015338.5:c.1934dup (p.Gly646Trpfs*12)	InDel	22.0
MDS_20	*BCOR*	NM_001123385.1:c.359C>G; p.(Pro120Arg)	Missense	52.0
MDS_20	*JAK2*	NM_004972.3:c.1972G>T; p.(Ala658Ser)	Missense	37.0
MDS_20	*RUNX1*	NM_001754.4:c.1167dup; p.(Gln390Alafs*210)	InDel	34.0
MDS_21	*ASXL1*	NM_015338.5:c.1900_1922del; p.(Glu635Argfs*15)	InDel	22.0
MDS_21	*CBL*	NM_005188.3:c.1249C>A; p.(Pro417Thr)	Missense	21.0
MDS_21	*ETNK1*	NM_018638.4:c.728A>T; p.(His243Leu)	Missense	18.0
MDS_21	*EZH2*	NM_004456.4:c.540delA; p.(Gln180Hisfs*61)	InDel	36.0
MDS_21	*TET2*	NM_001127208.2:c.3594+3A>C;p.?	Splice site	20.0
MDS_22	*SRSF2*	NM_003016.4:c.284C>T (p.Pro95Leu)	Missense	27.0
MDS_22	*TET2*	NM_001127208.2:c.4457C>G; p.(Ser1486*)	Nonsense	41.0

Subject	Gene	Variant	Mutation type	VAF [%]
MDS_22	*TET2*	NM_001127208.2:c.4138C>T; p.(His1380Tyr)	Missense	35.0
MDS_22	*ZRSR2*	NM_005089.3:c.491del; p.(Val164Glufs*74)	InDel	72.0
MDS_23	*SF3B1*	NM_012433.3:c.2098A>G, p.(Lys700Glu)	Missense	82.0
MDS_24	*DNMT3A*	NM_175629.2:c.2645G>C; p.(Arg882Pro)	Missense	32.0
MDS_24	*SF3B1*	NM_012433.3:c.1866G>T; p.(Glu622Asp)	Missense	36.0
MDS_24	*TET2*	NM_001127208.2:c.5618T>C; p.(Ile1873Thr)	Missense	16.0
MDS_25	*DNMT3A*	NM_175629.2:c.2206C>T; p.(Arg736Cys)	Missense	12.0
MDS_25	*RUNX1*	NM_001754.4:c.611G>A; p.(Arg204Gln)	Missense	38.0
MDS_25	*TET2*	NM_001127208.2:c.3415_3418del; p.(Ile1139Leufs*12)	InDel	3.1
MDS_25	*WT1*	NM_024426.5:c.1453C>T; p.[Arg485Trp]	Missense	40.0
MDS_26	*MPL*	NM_005373.2:c.1544G>T; p.(Trp515Leu)	Missense	56.0
MDS_26	*TET2*	NM_001127208.2:c.2713del; p.(Asp905Ilefs*16)	InDel	35.0
MDS_27	*RUNX1*	NM_001754.4:c.356_358dup; p.(Val119dup)	InDel	7.0
MDS_27	*TP53*	NM_000546.5:c.814G>A; p.(Val272Met)	Missense	13.0
MDS_27	*TP53*	NM_000546.5:c.273G>A; p.(Trp91*)	Nonsense	10.0
MDS_28	*ASXL1*	NM_015338.5:c.1934dup (p.Gly646Trpfs*12)	InDel	23.0
MDS_28	*KIT*	NM_000222.2:c.2447A>T; p.(Asp816Val)	Missense	12.0
MDS_28	*SETBP1*	NM_015559.3:c.2608G>A; p.(Gly870Ser)	Missense	6.0
MDS_28	*SETBP1*	NM_015559.3:c.2602G>A; p.(Asp868Asn)	Missense	3.0
MDS_28	*STAT3*	NM_139276.2:c.1846G>A; p.(Glu616Lys)	Missense	2.6
MDS_28	*TP53*	NM_000546.5:c.743G>A; p.(Arg248Gln)	Missense	7.0
MDS_29	*DNMT3A*	NM_175629.2:c.1093T>A; p.(Tyr365Asn)	Missense	4.0
MDS_29	*SRSF2*	NM_003016.4:c.284C>T; (p.Pro95Leu)	Missense	1.4
MDS_29	*TET2*	NM_001127208.2:c.4264A>T; p.(Lys1422*)	Nonsense	2.5
MDS_29	*TET2*	NM_001127208.2:c.4774dup; p.(Thr1592Asnfs*22)	InDel	2.0
MDS_30	*SF3B1*	NM_012433.3:c.2098A>G, p.(Lys700Glu)	Missense	45.0
MDS_30	*TET2*	NM_001127208.2:c.4109G>A; p.(Gly1370Glu)	Missense	30.0
MDS_30	*TET2*	NM_001127208.2:c.4211_4217del; p.(Arg1404Leufs*42)	InDel	23.0
MDS_31	*ASXL1*	NM_015338.5:c.1934dup; (p.Gly646Trpfs*12)	InDel	13.0
MDS_31	*RUNX1*	NM_001754.4:c.602G>A; p.(Arg201Gln)	Missense	28.0
MDS_31	*SRSF2*	NM_003016.4:c.284C>A; p.(Pro95His)	Missense	25.0
MDS_31	*STAG2*	NM_001042749.2:c.3034C>T; p.(Arg1012*)	Nonsense	31.0
MDS_32	*BCOR*	NM_001123385.1:c.2375del; p.(Asn792Ilefs*15)	InDel	4.7
MDS_32	*DNMT3A*	NM_175629.2:c.2098C>G; p.(Pro700Ala)	Missense	1.1
MDS_33	*SF3B1*	NM_012433.3:c.2098A>G; p.(Lys700Glu)	Missense	27.0
MDS_34	*ASXL2*	NM_018263.5:442C>T; p.(Gln148*)	Nonsense	35.0
MDS_34	*FLT3*	FLT3-ITD (66nt)	InDel	27.0
MDS_34	*SF3B1*	NM_012433.3:c.2098A>G, p.(Lys700Glu)	Missense	37.0
MDS_84	*MPL*	NM_005373.2:c.1654-10T>A	Splice site	15.3
MDS_35	*DNMT3A*	NM_175629.2:c.788_789dup; p.(Pro264Serfs*53)	InDel	33.0
MDS_35	*SF3B1*	NM_012433.3:c.2098A>G, p.(Lys700Glu)	Missense	37.0
MDS_35	*TET2*	NM_001127208.2:c.1389_1411dup; p.(Ser471Ilefs*23)	InDel	42.0
MDS_35	*TET2*	NM_001127208.2:c.2872_2873delCA; p.(Gln958Glufs*13)	InDel	36.0
MDS_35	*TET2*	NM_001127208.2:c.4075C>A; p.(Arg1359Ser)	Missense	6.3
MDS_36	*DNMT3A*	NM_175629.2:c.1851+5G>A;p.?	Splice site	3.8

Subject	Gene	Variant	Mutation type	VAF [%]
MDS_36	*TP53*	NM_000546.5:c.466_477dup; p.(Arg156_Ala159dup)	InDel	3.0
MDS_37	*DNMT3A*	NM_175629.2:c.1475-1G>C;p.?	Splice site	44.0
MDS_37	*SF3B1*	NM_012433.3:c.1972T>G; p.(Trp658Gly)	Missense	21.0
MDS_37	*SF3B1*	NM_012433.3:c.1997A>G; p.(Lys666Arg)	Missense	6.0
MDS_37	*SF3B1*	NM_012433.3:c.1973G>T; p.(Trp658Leu)	Missense	6.0
MDS_37	*SF3B1*	NM_012433.3:c.1866G>C; p.(Glu622Asp)	Missense	2.1
MDS_38	*RUNX1*	NM_001754.4:c.611G>A; p.(Arg204Gln)	Missense	6.0
MDS_38	*RUNX1*	NM_001754.4:c.602G>A; p.(Arg201Gln)	Missense	2.3
MDS_38	*RUNX1*	NM_001754.4:c.858_860dup; p.(Tyr287*)	InDel	1.5
MDS_38	*SH2B3*	NM_005475.2:c.1168delC; p.(Leu390Trpfs*21)	InDel	6.0
MDS_38	*SRSF2*	NM_003016.4:c.284C>G, p.(Pro95Arg)	Missense	21.0
MDS_38	*TET2*	NM_001127208.2:c.3646C>T; p.(Arg1216*)	Nonsense	23.0
MDS_38	*TET2*	NM_001127208.2:c.1454_1455delTG; p.(Val485Glufs*4)	InDel	15.0
MDS_38	*TP53*	NM_000546.5:c.646G>A; p.(Val216Met)	Missense	7.0
MDS_38	*TP53*	NM_000546.5:c.743G>A; p.(Arg248Gln)	Missense	2.2
MDS_39	*TET2*	NM_001127208.2:c.5500_5501delCA; p.(Gln1834Glyfs*11)	InDel	1.9
MDS_40	*U2AF1*	NM_001025203.1:c.101C>T; p.(Ser34Phe)	Missense	24.0
MDS_41	*DDX41*	NM_016222.4:c.209dup; p.(Arg71Profs*4)	InDel	39.0
MDS_41	*TERT*	ENST00000310581:c.3037C>T; p.[His1013Tyr]	Missense	1.3
MDS_42	*SF3B1*	NM_012433.3:c.2098A>G; p.(Lys700Glu)	Missense	46.4
MDS_42	*MPL*	NM_005373.2:c.1544G>C; p.(Trp515Ser)	Missense	8.0
MDS_43	*ASXL1*	NM_015338.5:c.1934dup; (p.Gly646Trpfs*12)	InDel	1.6
MDS_43	*DNMT3A*	NM_175629.2:c.976C>A; p.(Arg326Ser)	Missense	30.0
MDS_43	*DNMT3A*	NM_175629.2: c.2644C>T; p.(Arg882Cys)	Missense	1.5
MDS_43	*SF3B1*	NM_012433.3:c.2098A>G; p.(Lys700Glu)	Missense	32.0
MDS_43	*SH2B3*	NM_005475.2:c.1090T>C;p.(Trp364Arg)	Missense	2.8
MDS_44	*ASXL1*	NM_015338.5:c.1934dup; (p.Gly646Trpfs*12)	InDel	19.0
MDS_44	*IDH1*	NM_005896.3: c.395G>A; p.(Arg132His)	Missense	1.4
MDS_44	*IDH2*	NM_002168.3:c.419G>A; p.(Arg140Gln)	Missense	27.0
MDS_44	*SRSF2*	NM_003016.4:c.284C>G, p.(Pro95Arg)	Missense	29.0
MDS_44	*STAG2*	NM_001042749.2:c.3097C>T; p.(Arg1033*)	Nonsense	36.0
MDS_45	*SF3B1*	NM_012433.3:c.1997A>G; p.(Lys666Arg)	Missense	24.0
MDS_45	*ASXL1*	NM_015338.6:c.2827del;p.(Asp943Ilefs*2)	InDel	1.3
MDS_46	*DDX41*	NM_016222.4:c.1016C>T; p.(Pro339Leu)	Missense	15.0
MDS_47	*ASXL1*	NM_015338.6:c.1934dup p.(Gly646Trpfs*12)	InDel	13.0
MDS_47	*RUNX1*	NM_001754.4:c.931_934delACAA;p.(Thr311Profs*16)	InDel	12.3
MDS_48	*SF3B1*	NM_012433.3:c.2098A>G; p.(Lys700Glu)	Missense	32.0
MDS_49	*BCORL1*	NM_021946.4:c.[4619-1G>T];p.?	Splice site	83.0
MDS_49	*KRAS*	NM_033360.3:c.34G>A; p.(Gly12Ser)	Missense	1.1
MDS_49	*NRAS*	NM_002524.4:c.35G>C; p.(Gly12Ala)	Missense	24.0
MDS_49	*NRAS*	NM_002524.4:c.37G>C; p.(Gly13Arg)	Missense	7.5
MDS_49	*NRAS*	NM_002524.4:c.182A>G; p.(Gln61Arg)	Missense	1.1
MDS_49	*RUNX1*	NM_001754.4:c.664delT; p.(Ser222Profs*15)	InDel	43.0
MDS_49	*SF3B1*	NM_012433.3:c.2098A>G; p.(Lys700Glu)	Missense	43.0
MDS_49	*TET2*	NM_001127208.2:c.388delG; p.(Glu130Lysfs*15)	InDel	43.0

Subject	Gene	Variant	Mutation type	VAF [%]
MDS_50	*DNMT3A*	NM_175629.2:c.2309C>T; p.(Ser770Leu)	Missense	29.0
MDS_50	*SF3B1*	NM_012433.3:c.1984C>T; p.(His662Tyr)	Missense	33.0
MDS_51	*DNMT3A*	NM_175629.2: c.2645G>A; p.(Arg882His)	Missense	19.0
MDS_51	*DNMT3A*	NM_175629.2: c.1667+1G>A;p.?	Splice site	2.3
MDS_51	*SF3B1*	NM_012433.3:c.2098A>G; p.(Lys700Glu)	Missense	19.0
MDS_51	*TET2*	NM_001127208.2:c.1691G>A; p.(Trp564*)	Nonsense	2.3
MDS_51	*TET2*	NM_001127208.2:c.4263C>G; p.(Tyr1421*)	Nonsense	1.4
MDS_52	*ASXL1*	NM_015338.5:c.2290dup;p.(Leu764Profs*10)	InDel	1.3
MDS_52	*CBL*	NM_005188.3:c.1202G>C;p.(Cys401Ser)	Missense	1.3
MDS_52	*TET2*	NM_001127208.2:c.3764dup;p.(Tyr1255*)	InDel	87.0
MDS_53	*ASXL1*	NM_015338.5:c.1934dup;p.(Gly646Trpfs*12)	InDel	12.0
MDS_53	*MPL*	NM_005373.2:c.1775G>A;p.(Arg592Gln)	Missense	15.0
MDS_53	*U2AF1*	NM_001025203.1:c.470A>C;p.(Gln157Pro)	Missense	21.0
MDS_54	*ASXL1*	NM_015338.5:c.1819_1820delGG;p.(Gly607Leufs*11)	InDel	14.0
MDS_54	*SF3B1*	NM_012433.3:c.2342A>G:p.(Asp781Gly)	Missense	33.0
MDS_54	*TP53*	NM_000546.5:c.818G>A;p.(Arg273His)	Missense	34.0
MDS_55	*DNMT3A*	NM_175629.2:c.1429+1G>A;p.?	Splice site	1.5
MDS_56	*DNMT3A*	NM_175629.2:c.2645G>A;p.(Arg882His)	Missense	3.7
MDS_56	*SRSF2*	NM_003016.4:c.284_307delCCCCGGACTCACACCACAGCC GCC;p.(Pro95_Arg102del)	InDel	15.0
MDS_56	*TET2*	NM_001127208.2:c.3894dup;p.(Lys1299*)	InDel	16.0
MDS_57	*ASXL1*	NM_015338.5:c.2324T>G;p.(Leu775*)	Nonsense	1.1
MDS_57	*SF3B1*	NM_012433.3:c.1874G>T;p.(Arg625Leu)	Missense	6.4
MDS_57	*TET2*	NM_001127208.2:c.3658delA;p.(Thr1220Profs*6)	InDel	7.7
MDS_58	*RAD21*	NM_006265.3:c.1064C>T;p.(Pro355Leu)	Missense	21.4
MDS_58	*SRSF2*	NM_003016.4:c.284_307delCCCCGGACTCACACCACAGCC GCC;p.(Pro95_Arg102del)	InDel	23.4
MDS_58	*TET2*	NM_001127208.2:c.4044+1G>C;p.?	Splice site	10.0
MDS_59	*DNMT3A*	NM_175629.2:c.2612delC;p.(Pro871Glnfs*10)	InDel	3.7
MDS_59	*SF3B1*	NM_012433.3:c.1868A>G;p.(Tyr623Cys)	Missense	24.0
MDS_59	*SRSF2*	NM_003016.4:c.[283C>G];p.(Pro95Ala)	Missense	3.8
MDS_59	*TET2*	NM_001127208.2:c.3862G>C;p.(Gly1288Arg)	Missense	2.2
MDS_60	*RUNX1*	NM_001754.4:c.292delC;p.(Leu98Serfs*24)	InDel	1.7
MDS_60	*SRSF2*	NM_003016.4:c.284_307delCCCCGGACTCACACCACAGCC GCC;p.(Pro95_Arg102del)	InDel	26.0
MDS_60	*TET2*	NM_001127208.2:c.3921delG;p.(Lys1308Serfs*55)	InDel	1.8
MDS_60	*TET2*	NM_001127208.2:c.327delC;p.(Lys110Argfs*3)	InDel	4.0
MDS_61	*ASXL1*	NM_015338.5:c.1934dup;p.(Gly646Trpfs*12)	InDel	30.0
MDS_61	*BRAF*	NM_001354609.2:c.602A>G;p.(Gln201Arg)	Missense	44.0
MDS_61	*EZH2*	NM_004456.5:c.324delC;p.(Ile109*)	InDel	51.0
MDS_61	*FLT3*	NM_004119.3:c.2523C>A;p.(Asn841Lys)	Missense	40.0
MDS_61	*GATA2*	NM_001145661.2:c.229+1G>T;p.?	Splice site	40.0
MDS_61	*RUNX1*	NM_001754.4:c.[1207_1210dup]; p.(His404Leufs*197)	InDel	4.2
MDS_61	*RUNX1*	NM_001754.4:c.[951_952delTT];p.(Ser318Glnfs*281)	InDel	34.0
MDS_62	*ASXL1*	NM_015338.5:c.1934dup;p.(Gly646Trpfs*12)	InDel	12.3
MDS_62	*CEBPA*	NM_004364:c.514C>T;p.(Gln172*)	Nonsense	19.0

Subject	Gene	Variant	Mutation type	VAF [%]
MDS_62	*SF3B1*	NM_012433.3:c.1873C>T;p.(Arg625Cys)	Missense	15.0
MDS_62	*STAG2*	NM_001042750.2STAG2:c.1907dup;p.(Tyr636*)	InDel	30.0
MDS_62	*TET2*	NM_001127208.2:c.1813_1814delTA;p.(Tyr605Hisfs*32)	InDel	8.4
MDS_62	*TET2*	NM_001127208.2:c.5456delT;p.(Leu1819*)	InDel	18.0
MDS_63	*CBL*	NM_005188.3:c.1222T>G:p.(Trp408Gly)	Missense	16.3
MDS_63	*MPL*	NM_005373.3:c.545C>A; p.(Pro182His)	Missense	2.9
MDS_63	*NOTCH1*	ENST00000277541:c.7541_7542delCT:p.Pro2514fs	InDel	1.6
MDS_63	*TET2*	NM_001127208.2:c.4029_4030insC; p.(Ala1344Argfs*3)	InDel	34.6
MDS_64	*CBL*	NM_005188.3:c.1256G>A;p.(Cys419Tyr)	Missense	11.0
MDS_64	*EZH2*	NM_004456.5:c.2198A>G;p.(Tyr733Cys)	Missense	35.9
MDS_64	*TET2*	NM_001127208.2:c.5269delC;p.(His1757Ilefs*6)	InDel	19.7
MDS_65	*CSF3R*	NM_156039.3:c.2302C>T;p.(Gln768*)	Nonsense	2.5
MDS_65	*CSF3R*	NM_156039.3:c.2337T>A; p.(Tyr779*)	Nonsense	24.2
MDS_65	*DNMT3A*	NM_175629.2:c.2645G>A p.(Arg882His)	Missense	27.8
MDS_65	*U2AF1*	NM_001025203.1:c.470A>G;p.(Gln157Arg)	Missense	23.7
MDS_66	*TP53*	NM_000546.5:c.427G>A; p.(Val143Met)	Missense	27.4
MDS_67	*IDH1*	NM_005896.3: c.394C>T p.(Arg132Cys)	Missense	3.5
MDS_67	*RUNX1*	NM_001754.4:c.293T>C; p.(Leu98Pro)	Missense	30.3
MDS_67	*U2AF1*	NM_001025203.1:c.470A>C p.(Gln157Pro)	Missense	36.1
MDS_68	*SF3B1*	NM_012433.3:c.2098A>G p.(Lys700Glu)	Missense	39.0
MDS_69	*DNMT3A*	NM_175629.2:c.1851+1G>A; p.?	Splice site	4.7
MDS_69	*TET2*	NM_001127208.2:c.822del;p.(Asn275Ilefs*18)	InDel	3.1
MDS_69	*TP53*	NM_000546.5:c.473G>A;p.(Arg158His)	Missense	55.2
MDS_70	*ASXL1*	NM_015338.6:c.2535dup;p.(Ser846Glnfs*5)	InDel	33.0
MDS_70	*ETNK1*	NM_018638.4:c.731A>G;p.(Asn244Ser)	Missense	7.8
MDS_70	*U2AF1*	NM_001025203.1:c.470A>C p.(Gln157Pro)	Missense	6.9
MDS_70	*CALR*	NM_004343.3:c.1099_1150del;p.(Leu367Thrfs*46)	InDel	1.8
MDS_71	*ASXL1*	NM_015338.6:c.1804G>T;p.(Glu602*)	Nonsense	39.8
MDS_71	*CBL*	NM_005188.3:c.1151G>T;p.(Cys384Phe)	Missense	13.4
MDS_71	*ETNK1*	NM_018638.4:c.731A>G;p.(Asn244Ser)	Missense	1.7
MDS_71	*SETBP1*	NM_015559.3:c.2608G>A (p.Gly870Ser)	Missense	40.7
MDS_71	*SRSF2*	NM_003016.4:c.284C>T p.(Pro95Leu)	Missense	27.9
MDS_71	*ZRSR2*	NM_005089.3:c.1318_1319insCGGCC;p.(Arg440Profs*?)	InDel	36.0
MDS_72	*DNMT3A*	NM_175629.2:c.2628C>A;p.(Asp876Glu)	Missense	38.3
MDS_72	*DNMT3A*	NM_175629.2:c.2408+1G>A;p.?	Splice site	40.5
MDS_72	*SF3B1*	NM_012433.3:c.2098A>G p.(Lys700Glu)	Missense	40.9
MDS_72	*TET2*	NM_001127208.2:c.4115C>T;p.(Thr1372Ile)	Missense	39.8
MDS_73	*JAK2*	NM_004972.3:c.1849G>T p.(Val617Phe)	Missense	31.4
MDS_73	*TET2*	NM_001127208.2:c.2899C>T;p.(Gln967*)	Nonsense	40.5
MDS_73	*TET2*	NM_001127208.2:c.3594+4A>G;p.?	Splice site	42.3
MDS_73	*ZRSR2*	NM_005089.3:c.959G>A;p.(Cys320Tyr)	Missense	81.6
MDS_74	*EZH2*	NM_004456.5:c.2006G>A;p.(Ser669Asn)	Missense	83.7
MDS_74	*RUNX1*	NM_001754.4:c.602G>A;p.(Arg201Gln)	Missense	5.0
MDS_74	*SETBP1*	NM_015559.3:c.2602G>A p.(Asp868Asn)	Missense	31.7
MDS_74	*SF3B1*	NM_012433.3:c.1986C>G;p.(His662Gln)	Missense	42.7

**Subject**	**Gene**	**Variant**	**Mutation type**	**VAF [%]**
MDS_75	*DDX41*	NM_016222.2:c.1127C>T;p.(Ala376Val)	Missense	6.0
MDS_75	*DNMT3A*	NM_175629.2:c.2723A>C;p.(Tyr908Ser)	Missense	11.9
MDS_75	*TET2*	NM_001127208.2:c.3865T>G;p.(Cys1289Gly)	Missense	2.0
MDS_76	*DNMT3A*	NM_175629.2: c.2645G>A p.(Arg882His)	Missense	14.3
MDS_76	*DNMT3A*	NM_175629.2:c.2327A>G;p.(Asn776Ser)	Missense	17.9
MDS_77	*SF3B1*	NM_012433.3:c.2098A>G p.(Lys700Glu)	Missense	5.0
MDS_77	*RAD21*	NM_006265.3:c.415_419del;p.(Arg139Glyfs*19)	InDel	4.6
MDS_78	*TET2*	NM_001127208.2:c.1397del;p.(Ser466Leufs*20)	InDel	2.2
MDS_78	*TET2*	NM_001127208.2:c.4546C>T;p.(Arg1516*)	Nonsense	20.3
MDS_78	*TET2*	NM_001127208.2:c.3410-4A>G;p.?	Splice site	43.8
MDS_78	*DNMT3A*	NM_175629.2:c.1302del;p.(Val435Cysfs*216)	InDel	44.9
MDS_78	*SH2B3*	NM_005475.2:c.1153G>C;p.(Ala385Pro)	Missense	1.9
MDS_78	*JAK2*	NM_004972.3:c.1849G>T;p.(Val617Phe)	Missense	2.5
MDS_79	*TP53*	NM_000546.5:c.559+1G>T,p.?	Splice site	36.5
MDS_80	*SRSF2*	NM_003016.4:c.284C>A p.(Pro95His)	Missense	35.2
MDS_80	*ASXL1*	NM_015338.6:c.1904_1905del;p.(Glu635Glyfs*22)	InDel	37.6
MDS_80	*RUNX1*	NM_001754.4:c.998dup;p.(Arg334Alafs*266)	InDel	33.8
MDS_80	*KRAS*	NM_033360.4:c.437C>T p.(Ala146Val)	Missense	4.0
MDS_81	*JAK2*	NM_004972.3:c.1849G>T p.(Val617Phe)	Missense	5.0
MDS_81	*U2AF1*	NM_001025203.1:c.470A>G p.(Gln157Arg)	Missense	11.0
MDS_82	*TET2*	NM_001127208.2:c.3671del;p.(Ala1224Glufs*2)	InDel	1.8
MDS_82	*TP53*	NM_000546.5:c.659A>G;p.(Tyr220Cys)	Missense	18.5
MDS_82	*BRAF*	NM_004333.4:c.1406G>C;p.(Gly469Ala)	Missense	1.1
MDS_82	*ZBTB7A*	NM_015898.4:c.231del;p.(Ile77Metfs*43)	InDel	5.3
MDS_83	*SRSF2*	NM_003016.4:c.284C>A p.(Pro95His)	Missense	46.6
MDS_83	*ASXL1*	NM_015338.6:c.2182G>T;p.(Glu728*)	Nonsense	41.0
MDS_83	*STAG2*	NM_001042750.2:c.1840C>T p.(Arg614*)	Nonsense	86.0
AML_1	*ASXL1*	NM_015338:exon12:c.1927dupG:p.G642fs	InDel	11.6
AML_1	*BCOR*	NM_001123385:exon7:c.3446_3447del:p.A1149fs	InDel	63.9
AML_1	*BCOR*	NM_001123385:exon7:c.3444_3446G	InDel	35.2
AML_1	*CSF3R*	NM_156039:exon17:c.2436_2437insGCAG:p.S813fs	InDel	1.2
AML_1	*IDH2*	NM_002168:exon4:c.419G>A:p.R140Q	Missense	32.7
AML_1	*NRAS*	NM_002524:exon2:c.35G>T:p.G12V	Missense	18.9
AML_1	*PHF6*	NM_032458:exon9:c.954dupA:p.S318fs	InDel	5.9
AML_1	*RUNX1*	NM_001754:exon9:c.1141_1142insCGCC:p.L381fs	InDel	31.8
AML_1	*SRSF2*	NM_003016:exon1:c.284C>A:p.P95H	Missense	2.5
AML_1	*STAG2*	NM_001042750:exon15:c.1409delA:p.E470fs	InDel	61.0
AML_1	*TET2*	NM_001127208:exon3:c.2692G>T:p.G898X	Nonsense	4.0
AML_2	*ASXL1*	NM_015338:exon12:c.1927dupG:p.G642fs	InDel	18.4
AML_2	*CEBPA*	NM_004364:exon1:c.898C>T:p.R300C	Missense	25.1
AML_2	*RUNX1*	NM_001754:exon4:c.317G>T:p.W106L	Missense	33.8
AML_2	*STAG2*	NM_001042750:exon24:c.2308C>T:p.Q770X	Nonsense	27.6
AML_2	*U2AF1*	NM_001025203:exon6:c.470A>C:p.Q157P	Missense	28.8
AML_3	*ASXL1*	NM_015338:exon12:c.1927dupG:p.G642fs	InDel	15.2
AML_3	*BRAF*	NM_004333:c.1790T>A:p.L597Q	Missense	90.5

Subject	Gene	Variant	Mutation type	VAF [%]
AML_3	*EZH2*	NM_004456:exon19:c.2186T>C:p.F729S	Missense	92.3
AML_3	*IDH2*	NM_002168:exon4:c.419G>A:p.R140Q	Missense	46.4
AML_3	*RUNX1*	NM_001754:exon4:c.329A>C:p.K110T	Missense	3.1
AML_3	*STAG2*	NM_001042750:exon20:c.1840C>T:p.R614X	Nonsense	5.5
AML_3	*STAG2*	NM_001042750:exon21:c.2086_2096del:p.A696fs	InDel	7.9
AML_3	*STAG2*	NM_001042750:exon31:c.3394_3395insTA:p.L1132fs	InDel	27.9
AML_4	*ASXL1*	NM_015338:exon12:c.2077C>T:p.R693X	Nonsense	8.6
AML_4	*KRAS*	NM_033360:exon2:c.35G>A:p.G12D	Missense	38.3
AML_5	*FLT3*	NM_004119:exon20:c.2508_2510del:p.836_837del	InDel	7.2
AML_5	*U2AF1*	NM_001025203:exon2:c.101C>T:p.S34F	Missense	50.9
AML_6	*ASXL1*	NM_015338:exon12:c.2160delC:p.D720fs	InDel	42.9
AML_6	*RUNX1*	NM_001754:exon6:c.610C>T:p.R204X	Nonsense	38.6
AML_6	*SRSF2*	NM_003016:exon1:c.284C>A:p.P95H	Missense	41.9
AML_6	*TET2*	NM_001127208:exon3:c.757_770del:p.N253fs	InDel	40.0
AML_7	*IDH1*	NM_005896:exon4:c.394C>T:p.R132C	Missense	44.6
AML_7	*RUNX1*	NM_001754:exon5:c.369dupT:p.V124fs	InDel	47.2
AML_7	*SRSF2*	NM_003016:exon1:c.284C>T:p.P95L	Missense	40.4
AML_7	*TET2*	NM_001127208:exon11:c.5482C>T:p.Q1828X	Nonsense	99.0
AML_8	*BCOR*	NM_001123385:exon11:c.4428+2T>G,p.?	Splice site	50.8
AML_8	*BCORL1*	NM_021946:exon3:c.398_399insCGGT:p.S133fs	InDel	50.2
AML_8	*DNMT3A*	NM_175629:exon23:c.2644C>T:p.R882C	Missense	56.4
AML_8	*KIT*	NM_000222:exon8:c.1256_1261del:p.419_421del	InDel	41.3
AML_8	*RUNX1*	NM_001754:exon9:c.1007_1008insAGGGCCC:p.F336fs	InDel	41.0
AML_8	*SF3B1*	NM_012433:exon15:c.2098A>G:p.K700E	Missense	48.0
AML_9	*DNMT3A*	NM_175629:exon23:c.2645G>A:p.R882H	Missense	34.5
AML_9	*IDH2*	NM_002168:exon4:c.419G>A:p.R140Q	Missense	39.5
AML_9	*RUNX1*	NM_001754:exon9:c.1015_1016insCAGTTCCCCGCGC: p.L339fs	InDel	27.1
AML_10	*ETV6*	NM_001987:exon3:c.313_314insT:p.R105fs	InDel	37.7
AML_10	*FLT3*	NM_004119:exon14:c.1769_1770insTCAGCCCATCGGCTCCTCAGATAATGAGTACTT:p.F590delinsFQPIGSSDNEYF	InDel	25.3
AML_10	*NPM1*	NM_002520:exon10:c.805_806insGGGCGC:p.I269delinsRAL	InDel	37.9
AML_11	*DNMT3A*	NM_175629:exon13:c.1474+1G>A,p.?	Splice site	46.5
AML_11	*FLT3*	NM_004119:exon14:c.1799_1800insGTCCTCCTCAGATAATGAGTACTTCTACGTTGATTTCAGAGAATATGAATATGA:p.D600delinsESSSDNEYFYVDFREYEYD	InDel	1.2
AML_11	*RUNX1*	NM_001754:exon5:c.356dupT:p.V119fs	InDel	29.0
AML_11	*RUNX1*	NM_001754:exon4:c.299_300insATCGCAGAGGAGCAA:p.S100delinsSSQRSN	InDel	9.5
AML_11	*STAG2*	NM_001042750:exon28:c.2857C>T:p.R953X	Nonsense	26.2
AML_11	*U2AF1*	NM_001025203:exon2:c.101C>A:p.S34Y	Missense	52.4
AML_12	*ASXL1*	NM_015338:exon12:c.1888_1910del:p.H630fs	InDel	33.9
AML_12	*IDH2*	NM_002168:exon4:c.419G>A:p.R140Q	Missense	47.0
AML_12	*SRSF2*	NM_003016:exon1:c.284C>A:p.P95H	Missense	42.6
AML_12	*STAG2*	NM_001042750:exon20:c.1840C>T:p.R614X	Nonsense	14.6
AML_12	*STAG2*	NM_001042750:exon29:c.3034C>T:p.R1012X	Nonsense	65.2
AML_13	*NRAS*	NM_002524:exon2:c.38G>A:p.G13D	Missense	39.8

Subject	Gene	Variant	Mutation type	VAF [%]
AML_13	*SF3B1*	NM_012433:exon15:c.2098A>G:p.K700E	Missense	32.0
AML_14	*ASXL1*	NM_015338:exon12:c.1888_1910del:p.630_637del	InDel	43.7
AML_14	*BCOR*	NM_001123385:exon7:c.3490C>T:p.R1164X	Nonsense	94.2
AML_14	*DNMT3A*	NM_175629:exon18:c.2163G>C:p.K721N	Missense	89.3
AML_14	*ETV6*	NM_001987:exon3:c.164-2A>G,p.?	Splice site	44.5
AML_14	*NRAS*	NM_002524:exon2:c.35G>A:p.G12D	Missense	53.1
AML_14	*PTPN11*	NM_002834:exon3:c.181G>T:p.D61Y	Missense	12.9
AML_14	*RUNX1*	NM_001754:exon7:c.664delT:p.S222fs	InDel	39.5
AML_15	*KIT*	NM_000222:exon17:c.2447A>T:p.D816V	Missense	38.3
AML_15	*NRAS*	NM_002524:exon3:c.183A>C:p.Q61H	Missense	5.7
AML_15	*NRAS*	NM_002524:exon3:c.182A>G:p.Q61R	Missense	21.0
AML_15	*PTPN11*	NM_002834:exon3:c.226G>A:p.E76K	Missense	14.7
AML_15	*SF3B1*	NM_012433:exon15:c.2098A>G:p.K700E	Missense	44.7
AML_16	*CEBPA*	NM_004364:exon1:c.175G>T:p.E59X	Nonsense	76.7
AML_16	*CSF3R*	NM_156039:exon15:c.1919C>A:p.T640N	Missense	18.4
AML_16	*RUNX1*	NM_001754:exon9:c.1354G>A:p.V452M	Missense	57.5
AML_16	*SRSF2*	NM_003016:exon1:c.284C>A:p.P95H	Missense	41.6
AML_16	*STAG2*	NM_001042750:exon11:c.1015delA:p.K339fs	InDel	28.5
AML_16	*TET2*	NM_001127208:exon3:c.3315_3316del:p.I1105fs	InDel	44.1
AML_16	*TET2*	NM_001127208:exon6:c.3796A>G:p.N1266D	Missense	49.1
AML_16	*WT1*	NM_024426:exon7:c.1099-1G>A,p.?	Splice site	12.5
AML_17	*ASXL1*	NM_015338:exon12:c.2058_2059del:p.K686fs	InDel	45.3
AML_17	*BCOR*	NM_001123385:exon11:c.4540C>T:p.R1514X	Nonsense	38.9
AML_17	*NRAS*	NM_002524:exon2:c.35G>A:p.G12D	Missense	48.0
AML_17	*RUNX1*	NM_001754:exon4:c.245_246insAGCA:p.L82fs	InDel	56.0
AML_17	*SRSF2*	NM_003016:exon1:c.284C>A:p.P95H	Missense	44.4
AML_17	*STAG2*	NM_001042750:exon9:c.775C>T:p.R259X	Nonsense	45.1
AML_17	*TET2*	NM_001127208:exon5:c.3589A>G:p.K1197E	Missense	47.6
AML_18	*ASXL1*	NM_015338:exon12:c.1888_1910del:p.630_637del	InDel	25.8
AML_18	*IDH2*	NM_002168:exon4:c.515G>A:p.R172K	Missense	21.7
AML_18	*PTPN11*	NM_002834:exon13:c.1517A>C:p.Q506P	Missense	14.4
AML_18	*RUNX1*	NM_001754:exon6:c.610C>T:p.R204X	Nonsense	36.9
AML_18	*RUNX1*	NM_001754:exon6:c.592G>A:p.D198N	Missense	4.1
AML_18	*SRSF2*	NM_003016:exon1:c.284C>A:p.P95H	Missense	37.4
AML_18	*TET2*	NM_001127208:exon11:c.4834_4835del:p.1612_1612del	InDel	40.8
AML_19	*ASXL1*	NM_015338:exon12:c.2084delA:p.Q695fs	InDel	2.0
AML_19	*SRSF2*	NM_003016:exon1:c.284C>A:p.P95H	Missense	44.9
AML_19	*TET2*	NM_001127208:exon3:c.2218C>T:p.Q740X	Nonsense	48.3
AML_19	*TET2*	NM_001127208:exon7:c.3863G>A:p.G1288D	Missense	44.5
AML_20	*IDH2*	NM_002168:exon4:c.419G>A:p.R140Q	Missense	44.0
AML_21	*DNMT3A*	NM_175629:exon23:c.2645G>A:p.R882H	Missense	48.6
AML_22	*DNMT3A*	NM_175629:exon23:c.2644C>T:p.R882C	Missense	45.8
AML_22	*FLT3*	NM_004119:exon20:c.2503G>T:p.D835Y	Missense	7.6
AML_22	*RUNX1*	NM_001754:exon9:c.978_979insG:p.L327fs	InDel	46.1
AML_22	*SF3B1*	NM_012433:exon15:c.2098A>G:p.K700E	Missense	49.8

Subject	Gene	Variant	Mutation type	VAF [%]
AML_23	*GATA2*	NM_001145661:exon7:c.1160_1171del:p.387_391del	InDel	33.7
AML_23	*NRAS*	NM_002524:exon3:c.181C>A:p.Q61K	Missense	39.8
AML_24	*NPM1*	NM_002520:exon11:c.861_862insTGCA:p.L287fs	InDel	27.5
AML_24	*PTPN11*	NM_002834:exon3:c.179G>T:p.G60V	Missense	2.3
AML_24	*RAD21*	NM_006265:exon6:c.640C>T:p.Q214X	Nonsense	2.6
AML_25	*ASXL2*	NM_018263:exon11:c.1771_1772insCCCCACTGAGAATC:p.R591fs	InDel	47.2
AML_25	*FLT3*	NM_004119:exon15:c.1837+2->CGTGTTCAGAGAATATGAATATGATCTCAAATGGGAGTTTCCAAGAGAAAATTTAGAGTTTGG	Splice site	13.7
AML_25	*FLT3*	NM_004119:exon14:c.1790_1791insCTCACCTAACGATTTCAGAGAATA:p.Y597delinsYSPNDFREY	InDel	20.1
AML_25	*RUNX1*	NM_001754:exon8:c.861C>G:p.Y287X	Nonsense	45.0
AML_25	*SF3B1*	NM_012433:exon14:c.1996A>C:p.K666Q	Missense	38.4
AML_25	*WT1*	NM_024426:exon7:c.1137_1138insGAGGCGGGTC:p.R380fs	InDel	7.8
AML_26	*DNMT3A*	NM_175629:exon23:c.2645G>C:p.R882P	Missense	33.3
AML_26	*FLT3*	NM_004119:exon13:c.1613_1805GAGAATATGAATATGATCTCAAATGGGAGTT	InDel	15.7
AML_26	*FLT3*	NM_004119:exon14:c.1802_1803insTGATTTCAGAGAATATGAATATGATCT:p.L601delinsLDFREYEYDL	InDel	4.1
AML_26	*FLT3*	NM_004119:exon14:c.1800_1801insTTCAGAGAATATGAATATGAT:p.L601delinsFREYEYDL	InDel	23.9
AML_26	*NPM1*	NM_002520:exon11:c.859_860insTCTG:p.L287fs	InDel	38.4
AML_27	*KRAS*	NM_033360:exon2:c.38G>A:p.G13D	Missense	20.7
AML_27	*NPM1*	NM_002520:exon11:c.861_862insTGCA:p.L287fs	InDel	46.2
AML_28	*MPL*	NM_005373:exon10:c.1544G>T:p.W515L	Missense	2.2
AML_28	*U2AF1*	NM_001025203:exon6:c.470A>C:p.Q157P	Missense	20.4
AML_29	*DDX41*	NM_016222:exon15:c.1574G>A:p.R525H	Missense	7.8
AML_29	*TET2*	NM_001127208:exon7:c.3894T>A:p.C1298X	Nonsense	16.1
AML_30	*ETV6*	NM_001987:exon5:c.976_977insTTGGACCT:p.E326fs	InDel	0.6
AML_30	*WT1*	NM_024426:exon7:c.1138_1139insA:p.R380fs	InDel	22.5
AML_31	*BCOR*	NM_001123385:exon4:c.1005dupC:p.S336fs	InDel	44.3
AML_31	*BCORL1*	NM_021946:c.3001C>T:p.Q1001X	Nonsense	46.3
AML_31	*KRAS*	NM_033360:exon2:c.35G>A:p.G12D	Missense	42.4
AML_31	*RUNX1*	NM_001754:exon5:c.484A>G:p.R162G	Missense	27.9
AML_32	*CEBPA*	NM_004364:exon1:c.1000G>A:p.E334K	Missense	43.1
AML_32	*DNMT3A*	NM_175629:exon23:c.2645G>A:p.R882H	Missense	35.8
AML_32	*KRAS*	NM_033360:exon3:c.183A>T:p.Q61H	Missense	31.5
AML_32	*NPM1*	NM_002520:exon11:c.859_860insTCTG:p.L287fs	InDel	40.2
AML_32	*PTPN11*	NM_002834:exon3:c.182A>G:p.D61G	Missense	4.5
AML_32	*RAD21*	NM_006265:exon2:c.59C>T:p.A20V	Missense	3.7
AML_33	*BCOR*	NM_001123385:exon11:c.4428+1G>A,p.?	Splice site	86.6
AML_33	*KRAS*	NM_033360:exon2:c.37G>C:p.G13R	Missense	28.4
AML_33	*NRAS*	NM_002524:exon2:c.34G>A:p.G12S	Missense	12.4
AML_33	*RUNX1*	NM_001754:exon5:c.455dupA:p.K152fs	InDel	43.5
AML_33	*STAG2*	NM_001042750:exon20:c.1907_1908insTCCTC:p.Y636fs	InDel	85.4
AML_33	*TET2*	NM_001127208:exon11:c.5587G>A:p.A1863T	Missense	46.7
AML_34	*DNMT3A*	NM_175629:exon23:c.2644C>T:p.R882C	Missense	42.9

Subject	Gene	Variant	Mutation type	VAF [%]
AML_34	*DNMT3A*	NM_175629:exon18:c.2173G>A:p.E725K	Missense	42.4
AML_34	*JAK2*	NM_004972:exon14:c.1849G>T:p.V617F	Missense	14.9
AML_34	*SRSF2*	NM_003016:exon1:c.284C>A:p.P95H	Missense	38.1
AML_34	*TET2*	NM_001127208:exon7:c.3894dupT:p.C1298fs	InDel	81.9
AML_35	*NPM1*	NM_002520:exon11:c.859_860insTCTG:p.L287fs	InDel	36.8
AML_35	*STAG2*	NM_001042750:p.?	Splice site	70.8
AML_36	*TP53*	NM_000546:exon7:c.742C>T:p.R248W	Missense	96.3
AML_37	*BCOR*	NM_001123385:exon4:c.2962_2963insGT:p.S988fs	InDel	67.9
AML_37	*IDH2*	NM_002168:exon4:c.419G>A:p.R140Q	Missense	46.0
AML_37	*SRSF2*	NM_003016:exon1:c.284_307del:p.95_103del	InDel	41.2
AML_38	*BCOR*	NM_001123385:exon10:c.4425T>A:p.Y1475X	Nonsense	42.9
AML_38	*DNMT3A*	NM_175629:exon22:c.2510C>A:p.S837X	Nonsense	50.2
AML_38	*DNMT3A*	NM_175629:exon13:c.1490G>C:p.C497S	Missense	36.9
AML_38	*NPM1*	NM_002520:exon11:c.859_860insTCTG:p.L287fs	InDel	37.6
AML_38	*SF3B1*	NM_012433:exon15:c.2098A>G:p.K700E	Missense	42.8
AML_38	*TET2*	NM_001127208:exon3:c.2101C>T:p.Q701X	Nonsense	50.7
AML_38	*TET2*	NM_001127208:exon3:c.3249_3250A	InDel	2.7
AML_38	*TET2*	NM_001127208:exon10:c.4468G>T:p.E1490X	Nonsense	42.2
AML_39	*NRAS*	NM_002524:exon3:c.181C>A:p.Q61K	Missense	43.0
AML_39	*SF3B1*	NM_012433:exon15:c.2098A>G:p.K700E	Missense	49.5
AML_39	*WT1*	NM_024426:exon7:c.1144_1145insTCGG:p.A382fs	InDel	42.6
AML_40	*FLT3*	NM_004119:exon14:c.1809_1810insAGAGAATATGAATATGATCTCAAATGG:p.E604delinsREYEYDLKWE	InDel	8.9
AML_40	*FLT3*	NM_004119:exon14:c.1787_1788insCTTCTACGTTGATTTCAGAGA:p.E596delinsDFYVDFRE	InDel	14.4
AML_40	*FLT3*	NM_004119:exon14:c.1787_1788insGATGGTACAGGTGACCGGCTCCTCAGATAATGAGTACTTCTACGTTGATTTCAGAGA: p.E596delinsEMVQVTGSSDNEYFYVDFRE	InDel	24.4
AML_41	*SF3B1*	NM_012433:exon15:c.2098A>G:p.K700E	Missense	44.6
AML_41	*TP53*	NM_000546:exon4:c.325T>G:p.F109V	Missense	82.5
AML_41	*WT1*	NM_024426:exon1:c.332dupC:p.P111fs	InDel	46.0
AML_42	*BCOR*	NM_001123385:exon4:c.1882G>A:p.A628T	Missense	2.3
AML_42	*BCOR*	NM_001123385:exon4:c.1240G>A:p.A414T	Missense	3.2
AML_42	*FLT3*	NM_004119:exon15:c.1837+3->GATTTCAGAGAATATGAATATGATCTCAAATGGGAGTTTCCAAGAGAAAATTTAGAGTTTGGT	Splice site	37.3
AML_42	*STAG2*	NM_001042750:exon32:c.3468-3A>G,p.?	Splice site	2.1
AML_42	*TP53*	NM_000546:exon10:c.1004G>A:p.R335H	Missense	2.4
AML_42	*WT1*	NM_024426:exon7:c.1144_1145insTCGG:p.A382fs	InDel	43.5
AML_43	*PTPN11*	NM_002834:exon3:c.227A>G:p.E76G	Missense	21.7
AML_43	*RUNX1*	NM_001754:exon5:c.496C>T:p.R166X	Nonsense	37.1
AML_44	*CSF3R*	NM_156039:exon14:c.1853C>T:p.T618I	Missense	41.8
AML_44	*GATA2*	NM_001145661:exon7:c.1187G>T:p.R396L	Missense	39.4
AML_44	*NRAS*	NM_002524:exon3:c.182A>G:p.Q61R	Missense	16.2
AML_44	*SETBP1*	NM_015559:exon4:c.2608G>A:p.G870S	Missense	46.5
AML_44	*U2AF1*	NM_001025203:exon6:c.470A>C:p.Q157P	Missense	46.7

Subject	Gene	Variant	Mutation type	VAF [%]
AML_44	*WT1*	NM_024426:exon2:c.749dupT:p.M250fs	InDel	78.7
AML_45	*ASXL1*	NM_015338:exon12:c.1926dupA:p.G642fs	InDel	31.1
AML_45	*ETV6*	NM_001987:exon4:c.329-2A>G,p.?	Splice site	49.0
AML_45	*RUNX1*	NM_001754:exon7:c.676_677del:p.S226fs	InDel	46.7
AML_45	*SRSF2*	NM_003016:exon1:c.284C>G:p.P95R	Missense	49.0
AML_45	*TET2*	NM_001127208:exon3:c.2490dupA:p.I830fs	InDel	37.9
AML_46	*ASXL1*	NM_015338:exon12:c.1888_1910del:p.H630fs	InDel	36.1
AML_46	*FLT3*	NM_004119:exon14:c.1769_1770insGCCTCAGATAACCGGC TCCTCAGATAATGAGTACTT:p.F590delinsLPQITGSSDNEYF	InDel	69.4
AML_46	*GATA2*	NM_001145661:exon4:c.416_417del:p.S139fs	InDel	41.2
AML_46	*PTPN11*	NM_002834:exon3:c.215C>A:p.A72D	Missense	4.8
AML_46	*RUNX1*	NM_001754:exon7:c.623dupA:p.Q208fs	InDel	34.6
AML_46	*SRSF2*	NM_003016:exon1:c.284C>A:p.P95H	Missense	41.0
AML_46	*STAG2*	NM_001042750:exon13:c.1117-1->GATA,p.?	Splice site	12.5
AML_46	*STAG2*	NM_001042750:exon28:c.2857C>T:p.R953X	Nonsense	8.7
AML_46	*STAG2*	NM_001042750:exon28:c.2915dupT:p.M972fs	InDel	69.6
AML_47	*DDX41*	NM_016222:exon2:c.121C>T:p.Q41X	Nonsense	48.8
AML_48	*BCOR*	NM_001123385:exon12:c.4647_4649del:p.1549_1550del	InDel	17.9
AML_48	*IDH1*	NM_005896:exon4:c.394C>T:p.R132C	Missense	8.3
AML_48	*RUNX1*	NM_001754:p.?	Splice site	13.2
AML_48	*SRSF2*	NM_003016:exon1:c.284C>A:p.P95H	Missense	13.0
AML_48	*STAG2*	NM_001042750:exon10:c.868dupA:p.F289fs	InDel	7.5
AML_48	*WT1*	NM_024426:exon10:c.1462A>G:p.S488G	Missense	52.7
AML_49	*DNMT3A*	NM_175629:exon8:c.958C>T:p.R320X	Nonsense	32.9
AML_49	*IDH1*	NM_005896:exon4:c.394C>A:p.R132S	Missense	48.4
AML_49	*NPM1*	NM_002520:exon11:c.861_862insTGCA:p.L287fs	InDel	50.2
AML_50	*DNMT3A*	NM_175629:exon17:c.1936+3G>A,p.?	Splice site	66.2
AML_50	*TET2*	NM_001127208:exon11:c.5633G>A:p.R1878H	Missense	40.1
AML_50	*TP53*	NM_000546:exon6:c.596_598del:p.199_200del	InDel	4.3
AML_50	*TP53*	NM_000546:exon5:c.470T>A:p.V157D	Missense	83.3
AML_51	*BCOR*	NM_001123385:exon10:c.4288C>T:p.Q1430X	Nonsense	45.9
AML_51	*BCORL1*	NM_021946:c.3268C>T:p.R1090X	Nonsense	28.3
AML_51	*BCORL1*	NM_021946:c.3994C>T:p.R1332X	Nonsense	10.3
AML_51	*DNMT3A*	NM_175629:exon23:c.2645G>A:p.R882H	Missense	50.2
AML_51	*NRAS*	NM_002524:exon4:c.436G>A:p.A146T	Missense	3.4
AML_51	*RUNX1*	NM_001754:exon4:c.325A>T:p.N109Y	Missense	48.0
AML_51	*STAG2*	NM_001042750:exon28:c.2857C>T:p.R953X	Nonsense	48.3
AML_52	*TET2*	NM_001127208:exon3:c.1294G>T:p.E432X	Nonsense	53.0
AML_52	*TET2*	NM_001127208:exon8:c.3987delG:p.L1329fs	InDel	52.1
AML_53	*FLT3*	NM_004119:exon14:c.1787_1788insTCCCCACCAGCTACAG ATGGTACAGGTGACCGGCTCCTCAGATAATGAGTACTTCT ACGTTGATTTCAGAGA:p.E596delinsDPHQLQMVQVTGSSD NEYFYVDFRE	InDel	23.8
AML_54	*CBL*	NM_005188:exon8:c.1199T>G:p.M400R	Missense	79.0
AML_54	*IDH1*	NM_005896:exon4:c.395G>A:p.R132H	Missense	42.3
AML_54	*RUNX1*	NM_001754:exon6:c.611G>A:p.R204Q	Missense	8.7

Subject	Gene	Variant	Mutation type	VAF [%]
AML_54	*RUNX1*	NM_001754:exon5:c.370_371insGATG:p.V124fs	InDel	35.0
AML_54	*SRSF2*	NM_003016:exon1:c.284C>G:p.P95R	Missense	46.2
AML_55	*DNMT3A*	NM_175629:exon21:c.2409_2410insTAGG:p.P804_L805delinsX	Nonsense	53.9
AML_55	*RUNX1*	NM_001754:exon6:c.602G>A:p.R201Q	Missense	13.6
AML_55	*RUNX1*	NM_001754:exon5:c.496C>G:p.R166G	Missense	15.5
AML_55	*SF3B1*	NM_012433:exon15:c.2098A>G:p.K700E	Missense	28.3
AML_56	*ASXL1*	NM_015338:exon12:c.2925T>A:p.C975X	Nonsense	5.4
AML_56	*TP53*	NM_000546:exon6:c.659_672del:p.Y220fs	InDel	34.2
AML_57	*ASXL1*	NM_015338:exon12:c.1927dupG:p.G642fs	InDel	23.0
AML_57	*BCOR*	NM_001123385:exon4:c.466_467insCCCCCCAAAACACCGCCTGGAATAC:p.Q156fs	InDel	59.0
AML_57	*BCOR*	NM_001123385:exon4:c.464_465insCCCCCCCAAAACACCGCCTGGAAT:p.I155fs	InDel	5.7
AML_57	*RUNX1*	NM_001754:exon6:c.610C>T:p.R204X	Nonsense	20.5
AML_57	*SRSF2*	NM_003016:exon1:c.284C>A:p.P95H	Missense	42.5
AML_57	*STAG2*	NM_001042750:exon17:c.1535_1536AGC	InDel	15.4
AML_57	*STAG2*	NM_001042750:exon17:c.1536delA:p.A512fs	InDel	75.6
AML_58	*ASXL1*	NM_015338:exon12:c.2243delA:p.Q748fs	InDel	40.8
AML_58	*U2AF1*	NM_001025203:exon2:c.101C>T:p.S34F	Missense	47.3
AML_59	*ASXL1*	NM_015338:exon12:c.1888_1910del:p.H630fs	InDel	42.4
AML_59	*CBL*	NM_005188:exon8:c.1186T>C:p.C396R	Missense	29.0
AML_59	*CBL*	NM_005188:exon9:c.1250C>G:p.P417R	Missense	4.5
AML_59	*FLT3*	NM_004119:exon20:c.2503G>T:p.D835Y	Missense	32.0
AML_59	*SRSF2*	NM_003016:exon1:c.284C>A:p.P95H	Missense	48.5
AML_59	*TET2*	NM_001127208:exon3:c.1143delC:p.F381fs	InDel	46.2
AML_59	*TET2*	NM_001127208:exon3:c.1172delC:p.S391fs	InDel	45.7
AML_60	*BCORL1*	NM_021946:c.3304A>T:p.K1102X	Nonsense	89.1
AML_60	*ETV6*	NM_001987:exon3:c.305_306insGAAGGGGGACCTC:p.F102fs	InDel	23.1
AML_60	*FLT3*	NM_004119:exon14:c.1781_1782insGGT:p.F594delinsLV	InDel	26.7
AML_60	*NRAS*	NM_002524:exon3:c.183A>T:p.Q61H	Missense	2.5
AML_60	*NRAS*	NM_002524:exon2:c.37G>C:p.G13R	Missense	9.7
AML_61	*ASXL1*	NM_015338:exon12:c.2160delC:p.D720fs	InDel	48.4
AML_61	*IDH2*	NM_002168:exon4:c.418C>G:p.R140G	Missense	43.3
AML_61	*KRAS*	NM_033360:exon2:c.34G>C:p.G12R	Missense	3.3
AML_61	*NRAS*	NM_002524:exon2:c.35G>T:p.G12V	Missense	36.7
AML_61	*RUNX1*	NM_001754:exon7:c.694C>T:p.R232W	Missense	29.3
AML_61	*SRSF2*	NM_003016:exon1:c.284C>A:p.P95H	Missense	50.4
AML_61	*STAG2*	NM_001042750:exon20:c.1840C>T:p.R614X	Nonsense	94.7
AML_62	*FLT3*	NM_004119:exon20:c.2508_2510del:p.836_837del	InDel	2.2
AML_62	*IDH1*	NM_005896:exon4:c.395G>A:p.R132H	Missense	38.4
AML_63	*TP53*	NM_000546:exon8:c.818G>A:p.R273H	Missense	25.6
AML_63	*TP53*	NM_000546:exon6:c.657delC:p.P219fs	InDel	23.7
AML_64	*DNMT3A*	NM_175629:exon23:c.2645G>A:p.R882H	Missense	49.0
AML_64	*JAK2*	NM_004972:exon14:c.1849G>T:p.V617F	Missense	48.0
AML_64	*NRAS*	NM_002524:exon2:c.38G>T:p.G13V	Missense	48.0

Subject	Gene	Variant	Mutation type	VAF [%]
AML_64	*STAG2*	NM_001042750:exon6:c.328C>T:p.R110X	Nonsense	48.0
AML_64	*TET2*	NM_001127208:exon3:c.547dupA:p.L182fs	InDel	50.0
AML_65	*ASXL1*	NM_015338:exon12:c.4116_4117del:p.1372_1373del	InDel	44.7
AML_65	*NRAS*	NM_002524:exon2:c.38G>T:p.G13V	Missense	7.7
AML_65	*RUNX1*	NM_001754:exon5:c.492_493insTA:p.G165_R166delinsX	Nonsense	8.6
AML_65	*SRSF2*	NM_003016:exon1:c.283_284insGCC:p.P95delinsRP	InDel	47.0
AML_65	*STAG2*	NM_001042750:exon21:c.2096+1->T,p.?	Splice site	71.7
AML_65	*TET2*	NM_001127208:exon3:c.1452T>A:p.C484X	Nonsense	28.6
AML_65	*TET2*	NM_001127208:exon4:c.3430G>A:p.E1144K	Missense	48.9
AML_66	*ASXL1*	NM_015338:exon12:c.1888_1910del:p.H630fs	InDel	40.4
AML_66	*IDH1*	NM_005896:exon4:c.394C>G:p.R132G	Missense	45.2
AML_66	*JAK2*	NM_004972:exon14:c.1849G>T:p.V617F	Missense	3.4
AML_66	*RUNX1*	NM_001754:exon4:c.319C>T:p.R107C	Missense	44.3
AML_66	*SRSF2*	NM_003016:exon1:c.284C>A:p.P95H	Missense	50.8
AML_67	*TP53*	NM_000546:exon7:c.743G>A:p.R248Q	Missense	50.4
AML_68	*FLT3*	NM_004119:exon14:c.1834_1835insATGAATATGATCTCAAATGGGAGTTTCCAAGAGAAAATTTAGAGT:p.F612delinsYEYDLKWEFPRENLEF	InDel	12.0
AML_68	*FLT3*	NM_004119:exon14:c.1797_1798insCTTAACGTTGATTTCAGAGAATATGAATAT:p.D600delinsLNVDFREYEYD	InDel	4.9
AML_68	*FLT3*	NM_004119:exon14:c.1793_1794insCTACGTTGATTTCAGAGAATATGA:p.E598delinsDYVDFREYE	InDel	3.9
AML_68	*U2AF1*	NM_001025203:exon2:c.101C>T:p.S34F	Missense	44.6
AML_69	*FLT3*	NM_004119:exon15:c.1837+2->AAAGCGGGTGACCGGCTCCTCAGATAATGAGTACTTCTACGTTGATTTCAGAGAATATGAATATGATCTCAAATGGGAGTTTCCAAGAGAAAATTTAGAGTTTGG	Splice site	2.1
AML_69	*FLT3*	NM_004119:exon14:c.1815_1816insGGAAAGCGGGTGACCGGCTCCTCAGATAATGAGTACTTCTACGTTGATTTCAGAGAATATGAATATGATCTCAAATGGGAGTTT:p.P606delinsGKRVTGSSDNEYFYVDFREYEYDLKWEFP	InDel	11.8
AML_69	*FLT3*	NM_004119:exon14:c.1788_1789insGATAATGAGTACTTCTACGTTGATTTCAGAGAA:p.Y597delinsDNEYFYVDFREY	InDel	3.6
AML_69	*IDH2*	NM_002168:exon4:c.419G>A:p.R140Q	Missense	41.1
AML_69	*NPM1*	NM_002520:exon11:c.859_860insTCTG:p.L287fs	InDel	36.8
AML_69	*SRSF2*	NM_003016:exon1:c.284C>T:p.P95L	Missense	29.3
AML_69	*WT1*	NM_024426:exon7:c.1195A>C:p.N399H	Missense	47.4
AML_70	*BCOR*	NM_001123385:exon4:c.524_527del:p.175_176del	InDel	65.3
AML_70	*NRAS*	NM_002524:exon3:c.180_181insGCTTCC:p.Q61delinsASQ	InDel	15.3
AML_70	*RUNX1*	NM_001754:exon5:c.495_496insTT:p.R166fs	InDel	43.3
AML_70	*U2AF1*	NM_001025203:exon2:c.101C>A:p.S34Y	Missense	41.0
AML_71	*IDH2*	NM_002168:exon4:c.515G>A:p.R172K	Missense	35.0
AML_72	*BCOR*	NM_001123385:exon11:c.4428+1G>A,p.?	Splice site	36.2
AML_72	*DNMT3A*	NM_175629:exon23:c.2728G>C:p.A910P	Missense	38.2
AML_72	*DNMT3A*	NM_175629:exon8:c.942G>A:p.W314X	Nonsense	45.8
AML_72	*IDH2*	NM_002168:exon4:c.419G>A:p.R140Q	Missense	40.2
AML_73	*FLT3*	NM_004119:exon20:c.2503G>T:p.D835Y	Missense	3.8
AML_73	*NPM1*	NM_002520:exon11:c.859_860insTCTG:p.L287fs	InDel	37.0
AML_73	*NRAS*	NM_002524:exon2:c.38G>A:p.G13D	Missense	14.4

Subject	Gene	Variant	Mutation type	VAF [%]
AML_73	*PTPN11*	NM_002834:exon3:c.179G>T:p.G60V	Missense	3.3
AML_73	*RAD21*	NM_006265:exon8:c.933_934insTA:p.T312_V313delinsX	Nonsense	33.0
AML_74	*FLT3*	NM_004119:exon15:c.1837+1->ATTTCAGAGAATATGAATATGATCTCAAATGGGAGTTTCCAAGAGAAAATTTAGAGTTTG	Splice site	7.5
AML_74	*FLT3*	NM_004119:exon14:c.1778_1779insCCAGCTACAGATGGTACAGGTGACCGGCTCCTCAGATAATGAGTACTTCTACGTTGA:p.D593delinsDQLQMVQVTGSSDNEYFYVD	InDel	33.2
AML_74	*IDH2*	NM_002168:exon4:c.419G>A:p.R140Q	Missense	41.5
AML_75	*ASXL1*	NM_015338:exon12:c.1773C>A:p.Y591X	Nonsense	23.6
AML_75	*EZH2*	NM_004456:exon11:c.1262_1271del:p.T421fs	InDel	52.3
AML_75	*IDH1*	NM_005896:exon4:c.395G>A:p.R132H	Missense	34.5
AML_75	*PTPN11*	NM_002834:exon3:c.188A>G:p.Y63C	Missense	29.2
AML_75	*RUNX1*	NM_001754:exon9:c.1003_1013del:p.Q335fs	InDel	12.7
AML_75	*RUNX1*	NM_001754:exon6:c.508+1G>T,p.?	Splice site	15.7
AML_76	*DNMT3A*	NM_175629:exon23:c.2645G>A:p.R882H	Missense	35.5
AML_76	*KRAS*	NM_033360:exon2:c.35G>A:p.G12D	Missense	33.6
AML_76	*NPM1*	NM_002520:exon11:c.859_860insTCTG:p.L287fs	InDel	38.3
AML_77	*BCOR*	NM_001123385:exon8:c.3649C>T:p.R1217X	Nonsense	16.3
AML_77	*DNMT3A*	NM_175629:exon8:c.912delC:p.S304fs	InDel	28.3
AML_77	*IDH2*	NM_002168:exon4:c.419G>A:p.R140Q	Missense	23.4
AML_78	*BCOR*	NM_001123385:exon10:c.4174_4225del:p.V1392fs	InDel	4.1
AML_78	*BCORL1*	NM_021946:c.2843_2844del:p.Q948fs	InDel	86.6
AML_78	*NRAS*	NM_002524:exon2:c.38G>A:p.G13D	Missense	42.9
AML_78	*RUNX1*	NM_001754:exon9:c.1290dupG:p.P431fs	InDel	43.2
AML_78	*U2AF1*	NM_001025203:exon2:c.101C>T:p.S34F	Missense	47.8
AML_79	*DDX41*	NM_016222:exon15:c.1586_1587del:p.T529fs	InDel	46.8
AML_79	*DNMT3A*	NM_175629:exon13:c.1502A>G:p.N501S	Missense	54.1
AML_79	*EZH2*	NM_004456:exon17:c.1973_1974del:p.R658fs	InDel	60.5
AML_80	*ASXL1*	NM_015338:exon12:c.2084dupA:p.Q695fs	InDel	39.1
AML_80	*RUNX1*	NM_001754:exon4:c.230dupG:p.S77fs	InDel	45.6
AML_80	*SRSF2*	NM_003016:exon1:c.284C>A:p.P95H	Missense	40.6
AML_80	*STAG2*	NM_001042750:exon27:c.2694_2695insTGGTAGAGTTGA:p.D898delinsDWX	Nonsense	29.4
AML_81	*ASXL1*	NM_015338:exon12:c.1927dupG:p.G642fs	InDel	41.4
AML_81	*EZH2*	NM_004456:exon18:c.2050C>T:p.R684C	Missense	9.9
AML_81	*NRAS*	NM_002524:exon2:c.38G>A:p.G13D	Missense	42.1
AML_81	*RUNX1*	NM_001754:exon6:c.553C>A:p.Q185K	Missense	42.3
AML_81	*SRSF2*	NM_003016:exon1:c.284C>A:p.P95H	Missense	50.0
AML_81	*STAG2*	NM_001042750:exon30:c.3097C>T:p.R1033X	Nonsense	73.5
AML_82	*ASXL1*	NM_015338:exon12:c.1927dupG:p.G642fs	InDel	15.4
AML_82	*PHF6*	NM_032458:exon6:c.442G>T:p.E148X	Nonsense	32.9
AML_82	*U2AF1*	NM_001025203:exon2:c.101C>T:p.S34F	Missense	30.1
AML_83	*EZH2*	NM_004456:exon19:c.2161A>G:p.I721V	Missense	54.0
AML_83	*FLT3*	NM_004119:exon20:c.2503G>T:p.D835Y	Missense	13.4
AML_83	*FLT3*	NM_004119:exon14:c.1796_1796delinsCCCTAGATAATGAGTACTTCTACGTTGATTTCAGAGAATATGAAT	InDel	0.5

Subject	Gene	Variant	Mutation type	VAF [%]
AML_83	*NPM1*	NM_002520:exon11:c.859_860insTCTG:p.L287fs	InDel	36.3
AML_83	*PTPN11*	NM_002834:exon3:c.179G>C:p.G60A	Missense	6.8
AML_83	*WT1*	NM_024426:exon7:c.1142C>A:p.S381X	Nonsense	17.2
AML_83	*WT1*	NM_024426:exon1:c.252_256del:p.84_86del	InDel	33.3
AML_84	*ASXL1*	NM_015338:exon12:c.1927dupG:p.G642fs	InDel	27.0
AML_84	*BCOR*	NM_001123385:exon11:c.4455_4456insGG:p.K1486fs	InDel	80.9
AML_84	*BCOR*	NM_001123385:exon11:c.4455_4455delinsTGG	InDel	86.9
AML_84	*EZH2*	NM_004456:exon18:c.2051G>A:p.R684H	Missense	5.2
AML_84	*HNRNPK*	NM_002140:exon16:c.1192-2A>G,p.?	Splice site	40.6
AML_84	*STAG2*	NM_001042750:exon19:c.1810C>T:p.R604X	Nonsense	88.2
AML_85	*ASXL1*	NM_015338:exon12:c.1927dupG:p.G642fs	InDel	23.6
AML_85	*GATA1*	NM_002049:exon3:c.325G>C:p.V109L	Missense	92.5
AML_85	*IDH2*	NM_002168:exon4:c.419G>A:p.R140Q	Missense	46.2
AML_85	*SRSF2*	NM_003016:exon1:c.284C>A:p.P95H	Missense	48.8
AML_85	*STAG2*	NM_001042750:exon20:c.1907dupA:p.Y636_H637delinsX	Nonsense	91.9
AML_86	*DNMT3A*	NM_175629:exon20:c.2322+1G>A,p.?	Splice site	37.7
AML_86	*NPM1*	NM_002520:exon11:c.859_860insTCTG:p.L287fs	InDel	51.5
AML_86	*NRAS*	NM_002524:exon2:c.34G>A:p.G12S	Missense	4.1
AML_86	*PTPN11*	NM_002834:exon3:c.179G>T:p.G60V	Missense	36.9
AML_86	*TET2*	NM_001127208:exon6:c.3707T>A:p.I1236N	Missense	2.7
AML_87	*KRAS*	NM_033360:exon2:c.35G>T:p.G12V	Missense	5.1
AML_87	*NRAS*	NM_002524:exon2:c.35G>A:p.G12D	Missense	6.8
AML_87	*TET2*	NM_001127208:exon11:c.4883_4884insTCCATCATATCAAT GCAATGGAAACC:p.Y1628fs	InDel	13.9
AML_88	*DNMT3A*	NM_175629:exon14:c.1627G>T:p.G543C	Missense	12.1
AML_88	*FLT3*	NM_004119:exon15:c.1837+10>AGAATAATGAGTACTTCTA CGTTGATTTCAGAGAATATGAATATGATCTCAAATGGGAG TTTCCAAGAGAAAATTTAGAGTTTG	InDel	1.6
AML_88	*NPM1*	NM_002520:exon11:c.859_860insTCTG:p.L287fs	InDel	20.4
AML_88	*WT1*	NM_024426:exon7:c.1144_1145insTCGG:p.A382fs	InDel	6.2
AML_89	*ASXL1*	NM_015338:exon12:c.2077C>T:p.R693X	Nonsense	8.3
AML_89	*FLT3*	NM_004119:exon14:c.1808_1808delinsTCAGAGAATATGAAT ATGATCTCAAAT	InDel	0.3
AML_89	*NRAS*	NM_002524:exon3:c.182A>C:p.Q61P	Missense	4.1
AML_89	*NRAS*	NM_002524:exon2:c.38G>T:p.G13V	Missense	39.1
AML_89	*RUNX1*	NM_001754:exon5:c.374delC:p.P125fs	InDel	46.0
AML_89	*SRSF2*	NM_003016:exon1:c.284C>A:p.P95H	Missense	44.6
AML_89	*STAG2*	NM_001042750:exon9:c.687dupT:p.A229fs	InDel	94.9
AML_89	*TET2*	NM_001127208:exon11:c.5072_5073insTA:p.S1691fs	InDel	46.9
AML_90	*ASXL1*	NM_015338:exon12:c.1927dupG:p.G642fs	InDel	19.5
AML_90	*TET2*	NM_001127208:exon10:c.4210C>T:p.R1404X	Nonsense	17.1
AML_91	*JAK2*	NM_004972:exon14:c.1849G>T:p.V617F	Missense	98.6
AML_92	*DNMT3A*	NM_175629:exon16:c.1898C>A:p.P633H	Missense	47.4
AML_92	*IDH2*	NM_002168:exon4:c.419G>A:p.R140Q	Missense	6.4
AML_92	*NPM1*	NM_002520:exon11:c.859_860insTCTG:p.L287fs	InDel	43.1
AML_92	*NRAS*	NM_002524:exon2:c.38G>A:p.G13D	Missense	4.3

Subject	Gene	Variant	Mutation type	VAF [%]
AML_92	*NRAS*	NM_002524:exon2:c.35G>A:p.G12D	Missense	37.1
AML_92	*RAD21*	NM_006265:exon13:c.1621-1G>T,p.?	Splice site	39.9
AML_93	*DNMT3A*	NM_175629:exon19:c.2311C>T:p.R771X	Nonsense	51.0
AML_93	*DNMT3A*	NM_175629:exon15:c.1687G>A:p.V563M	Missense	52.1
AML_93	*IDH2*	NM_002168:exon4:c.419G>A:p.R140Q	Missense	48.2
AML_93	*JAK2*	NM_004972:exon14:c.1849G>T:p.V617F	Missense	47.6
AML_93	*SRSF2*	NM_003016:exon1:c.284C>A:p.P95H	Missense	40.1
AML_94	*ASXL1*	NM_015338:exon12:c.1888_1910del:p.630_637del	InDel	21.5
AML_94	*ASXL1*	NM_015338:exon12:c.2118_2119insAC:p.H706fs	InDel	8.9
AML_94	*DNMT3A*	NM_175629:exon19:c.2255_2257del:p.752_753del	InDel	47.5
AML_94	*DNMT3A*	NM_175629:exon19:c.2206C>T:p.R736C	Missense	47.6
AML_94	*IDH2*	NM_002168:exon4:c.515G>A:p.R172K	Missense	45.4
AML_95	*NRAS*	NM_002524:exon3:c.182A>G:p.Q61R	Missense	11.2
AML_95	*TP53*	NM_000546:exon8:c.844C>T:p.R282W	Missense	63.0
AML_95	*TP53*	NM_000546:exon4:c.97-2A>G,p.?	Splice site	20.0
AML_96	*DNMT3A*	NM_175629:exon23:c.2645G>A:p.R882H	Missense	38.5
AML_96	*NPM1*	NM_002520:exon11:c.859_860insTCTG:p.L287fs	InDel	36.5
AML_96	*NRAS*	NM_002524:exon2:c.35G>A:p.G12D	Missense	37.9
AML_96	*U2AF1*	NM_001025203:exon2:c.101C>T:p.S34F	Missense	41.6
AML_97	*DNMT3A*	NM_175629:exon23:c.2711C>T:p.P904L	Missense	18.7
AML_97	*IDH2*	NM_002168:exon4:c.419G>A:p.R140Q	Missense	19.6
AML_97	*KRAS*	NM_033360:exon2:c.35G>A:p.G12D	Missense	2.9
AML_98	*NPM1*	NM_002520:exon11:c.859_860insTCTG:p.L287fs	InDel	31.0
AML_98	*TET2*	NM_001127208:exon3:c.1441C>T:p.Q481X	Nonsense	34.1
AML_98	*TET2*	NM_001127208:exon3:c.2491C>T:p.Q831X	Nonsense	56.3
AML_99	*DNMT3A*	NM_175629:exon16:c.1906G>T:p.V636L	Missense	96.3
AML_99	*FLT3*	NM_004119:exon14:c.1810_1811insTAGTTGATTTCAGAGAATATGAATATGATCTCAAATGGG:p.E604delinsVVDFREYEYDLKWE	InDel	29.4
AML_99	*FLT3*	NM_004119:exon14:c.1792_1793insTCGATTTCAGAGAATATG:p.E598delinsVDFREYE	InDel	12.7
AML_99	*RUNX1*	NM_001754:exon4:c.330_331insT:p.T111fs	InDel	22.8
AML_100	*ETV6*	NM_001987:exon6:c.1138T>G:p.W380G	Missense	25.0
AML_100	*KRAS*	NM_033360:exon3:c.183A>C:p.Q61H	Missense	14.9
AML_100	*WT1*	NM_024426:exon9:c.1395C>A:p.H465Q	Missense	5.4
AML_100	*WT1*	NM_024426:exon7:c.1199dupA:p.K400fs	InDel	3.1
AML_101	*ASXL1*	NM_015338:exon12:c.1927dupG:p.G642fs	InDel	23.2
AML_101	*KDM6A*	NM_021140:exon13:c.1310C>T:p.A437V	Missense	2.5
AML_101	*RUNX1*	NM_001754:exon9:c.989dupT:p.F330fs	InDel	39.0
AML_101	*TET2*	NM_001127208:exon7:c.3866G>T:p.C1289F	Missense	36.4
AML_101	*U2AF1*	NM_001025203:exon6:c.467G>A:p.R156H	Missense	25.5
AML_102	*BCOR*	NM_001123385:exon13:c.4742-1G>T,p.?	Splice site	25.0
AML_102	*NRAS*	NM_002524:exon2:c.34G>T:p.G12C	Missense	13.5
AML_102	*RUNX1*	NM_001754:exon6:c.509-3C>G,p.?	Splice site	58.8
AML_102	*SF3B1*	NM_012433:exon15:c.2098A>G:p.K700E	Missense	52.4
AML_102	*TET2*	NM_001127208:exon6:c.3641G>A:p.R1214Q	Missense	20.0

Subject	Gene	Variant	Mutation type	VAF [%]
AML_103	*ASXL1*	NM_015338:exon12:c.1931_1932insGGGT:p.G644fs	InDel	23.9
AML_103	*RUNX1*	NM_001754:exon5:c.496C>T:p.R166X	Nonsense	42.1
AML_103	*SRSF2*	NM_003016:exon1:c.284C>A:p.P95H	Missense	39.5
AML_103	*TET2*	NM_001127208:exon3:c.1937_1938insAGTGG:p.T646fs	InDel	28.0
AML_103	*TET2*	NM_001127208:exon6:c.3782G>A:p.R1261H	Missense	23.7
AML_104	*RUNX1*	NM_001754:exon6:c.508+1G>A,p.?	Splice site	36.3
AML_104	*RUNX1*	NM_001754:exon5:c.508G>C:p.G170R	Missense	26.8
AML_104	*RUNX1*	NM_001754:exon5:c.422C>A:p.S141X	Nonsense	4.3
AML_104	*SF3B1*	NM_012433:exon15:c.2098A>G:p.K700E	Missense	40.8
AML_105	*DNMT3A*	NM_175629:exon23:c.2645G>A:p.R882H	Missense	43.6
AML_105	*KRAS*	NM_033360:exon3:c.176C>G:p.A59G	Missense	4.7
AML_105	*NPM1*	NM_002520:exon11:c.859_860insTCTG:p.L287fs	InDel	47.0
AML_105	*PTPN11*	NM_002834:exon3:c.182A>G:p.D61G	Missense	27.5
AML_105	*PTPN11*	NM_002834:exon3:c.213T>A:p.F71L	Missense	6.0
AML_106	*PTPN11*	NM_002834:exon3:c.181G>T:p.D61Y	Missense	10.2
AML_106	*TP53*	NM_000546:exon6:c.646G>A:p.V216M	Missense	82.6
AML_107	*NPM1*	NM_002520:exon11:c.859_860insTCTG:p.L287fs	InDel	20.2
AML_107	*NRAS*	NM_002524:exon2:c.35G>A:p.G12D	Missense	3.3
AML_107	*SRSF2*	NM_003016:exon1:c.284C>T:p.P95L	Missense	25.6
AML_107	*TET2*	NM_001127208:exon7:c.3852_3854del:p.1284_1285del	InDel	37.7
AML_107	*TET2*	NM_001127208:exon10:c.4393C>T:p.R1465X	Nonsense	46.1
AML_108	*DNMT3A*	NM_175629:exon23:c.2678G>C:p.W893S	Missense	39.4
AML_108	*FLT3*	NM_004119:exon14:c.1831_1832insTTGATTTCAGAGAATAT GAATATGATCTCAAATGGGAGTTTCCAAGAGAAAATTTAG: p.E611delinsVDFREYEYDLKWEFPRENLE	InDel	22.8
AML_108	*NPM1*	NM_002520:exon11:c.859_860insTCTG:p.L287fs	InDel	46.3
AML_109	*DNMT3A*	NM_175629:exon23:c.2645G>A:p.R882H	Missense	44.0
AML_109	*FLT3*	NM_004119:exon14:c.1816_1817insAAAATGAGTACTTCTAC GTTGATTTCAGAGAATATGAATATGATCTCAAATGGGAGTT TC:p.P606delinsQNEYFYVDFREYEYDLKWEFP	InDel	5.6
AML_109	*FLT3*	NM_004119:exon14:c.1793_1794insCTACGTTGATTTCAGA GAATATGA:p.E598delinsDYVDFREYE	InDel	3.0
AML_109	*FLT3*	NM_004119:exon14:c.1784_1785insCCCTTATTTCAG:p.R595 delinsSPYFR	InDel	2.9
AML_109	*FLT3*	NM_004119:exon14:c.1783_1784insCCTCAGATAATGAGTA CTTCTACGTTGATTTCA:p.R595delinsTSDNEYFYVDFR	InDel	1.5
AML_109	*KRAS*	NM_033360:exon2:c.35G>A:p.G12D	Missense	7.5
AML_109	*NPM1*	NM_002520:exon11:c.859_860insTCTG:p.L287fs	InDel	39.3
AML_109	*NRAS*	NM_002524:exon2:c.38G>A:p.G13D	Missense	6.1
AML_109	*PTPN11*	NM_002834:exon3:c.205G>A:p.E69K	Missense	3.9
AML_109	*WT1*	NM_024426:exon7:c.1140_1141insCCGG:p.S381fs	InDel	6.4
AML_110	*ASXL1*	NM_015338:exon12:c.1927dupG:p.G642fs	InDel	21.0
AML_110	*PTPN11*	NM_002834:exon13:c.1529A>T:p.Q510L	Missense	23.2
AML_110	*RUNX1*	NM_001754:exon7:c.720_733del:p.240_245del	InDel	53.0
AML_110	*SRSF2*	NM_003016:exon1:c.284C>T:p.P95L	Missense	17.4
AML_110	*STAG2*	NM_001042750:exon5:c.265dupA:p.V88fs	InDel	37.8
AML_110	*STAG2*	NM_001042750:exon5:c.269_269delinsAG	InDel	28.3
AML_110	*STAG2*	NM_001042750:exon5:c.269T>G:p.M90R	Missense	38.5

Subject	Gene	Variant	Mutation type	VAF [%]
AML_111	*NPM1*	NM_002520:exon11:c.859_860insTCTG:p.L287fs	InDel	37.8
AML_111	*PTPN11*	NM_002834:exon3:c.179G>T:p.G60V	Missense	44.2
AML_111	*STAG2*	NM_001042750:exon17:c.1535-3T>A,p.?	Splice site	47.8
AML_112	*BCOR*	NM_001123385:exon4:c.1555_1559del:p.E519fs	InDel	80.7
AML_112	*FLT3*	NM_004119:exon14:c.1794_1795insAAGGTGACCGGCTCCTCAGATAATGAGTACTTCTACGTTGATTTCAGAGAATATGAA:p.Y599delinsKVTGSSDNEYFYVDFREYEY	InDel	16.5
AML_112	*NRAS*	NM_002524:exon3:c.176C>A:p.A59D	Missense	14.1
AML_112	*STAG2*	NM_001042750:exon9:c.705_706insT:p.L235fs	InDel	79.1
AML_112	*STAG2*	NM_001042750:exon9:c.706_706delinsTG	InDel	8.9
AML_112	*U2AF1*	NM_001025203:exon2:c.101C>T:p.S34F	Missense	42.8
AML_113	*ASXL1*	NM_015338:exon12:c.1888_1910del:p.H630fs	InDel	39.5
AML_113	*KRAS*	NM_033360:exon2:c.64C>A:p.Q22K	Missense	10.9
AML_113	*SRSF2*	NM_003016:exon1:c.284C>T:p.P95L	Missense	20.6
AML_113	*TET2*	NM_001127208:exon3:c.1110T>A:p.Y370X	Nonsense	68.4
AML_114	*DNMT3A*	NM_175629:exon23:c.2645G>A:p.R882H	Missense	48.0
AML_114	*FLT3*	NM_004119:exon14:c.1793_1794insCTTCGAAAGCCAGCTACAGATGGTACAGGTGACCGGCTCCTCAGATAATGAGTACTTCTACGTTGATTTCAGAGAATATGA:p.E598delinsDFESQLQMVQVTGSSDNEYFYVDFREYE	InDel	9.0
AML_114	*NPM1*	NM_002520:exon11:c.859_860insTCTG:p.L287fs	InDel	43.0
AML_114	*WT1*	NM_024426:exon7:c.1142_1143insAACAAGAGTC:p.S381fs	InDel	25.0
AML_115	*ASXL1*	NM_015338:exon12:c.2077C>T:p.R693X	Nonsense	16.0
AML_115	*CEBPA*	NM_004364:exon1:c.961_962insC:p.N321fs	InDel	65.0
AML_115	*CEBPA*	NM_004364:exon1:c.178dupA:p.T60fs	InDel	34.0
AML_115	*SRSF2*	NM_003016:exon1:c.284C>T:p.P95L	Missense	19.0
AML_115	*STAG2*	NM_001042750:exon10:c.891C>A:p.Y297X	Nonsense	94.0
AML_116	*DNMT3A*	NM_175629:exon19:c.2196T>G:p.F732L	Missense	43.6
AML_116	*FLT3*	NM_004119:exon14:c.1808_1809insTCCAGATAATGAGTACTTCTACGTTGATTTCAGAGAATATGAATATGATCTCAAATG:p.W603delinsCPDNEYFYVDFREYEYDLKW	InDel	27.7
AML_116	*NPM1*	NM_002520:exon11:c.860_861insCTGC:p.L287fs	InDel	33.6
AML_116	*WT1*	NM_024426:exon7:c.1142C>A:p.S381X	Nonsense	46.1
AML_117	*IDH1*	NM_005896:exon4:c.394C>T:p.R132C	Missense	18.6

Abbreviations: VAF, variant allele frequency; InDel, insertion/deletion variant; MDS, myelodysplastic neoplasm; AML, acute myeloid leukemia.

Supplemental Table 12: Summary statistics of mutations identified in individuals undergoing total hip arthroplasty and patients with MDS or sAML.

	**THA (n=261)**	**MDS (n=91)**	**sAML (n=123)**
Individuals with ≥1 somatic variant	127/261 (49%)	84/91 (92%)	117/123 (95%)
Variants per individual (range)	0 - 5	0 - 9	0 - 11
Among individuals with ≥1 variant:			
- Median number of variants	1	3	4
- VAF [%] (median, range)	2.7 (1 - 44)	19 (1 - 87)	37 (0.3 - 99)

Abbreviations: VAF, variant allele frequency; THA, total hip arthroplasty; MDS, myelodysplastic neoplasm; AML, acute myeloid leukemia.

Supplemental Table 13: Comparison of *DNMT3A*, *TET2* and *ASXL1* variant properties between CH, MDS and sAML.

	CH (n=127)	MDS (n=91)	sAML (n=123)
DNMT3A p.R882 vs. others	4/87 (5%)	9/28 (32%)	14/37 (38%)
TET2 InDel or nonsense vs. others	27/47 (57%)	43/62 (69%)	27/37 (73%)
ASXL1 c.1934dup vs. others	3/12 (25%)	9/22 (41%)	12/34 (35%)

Abbreviations: InDel, insertion/deletion variant; MDS, myelodysplastic neoplasm; AML, acute myeloid leukemia.

Supplemental Figures

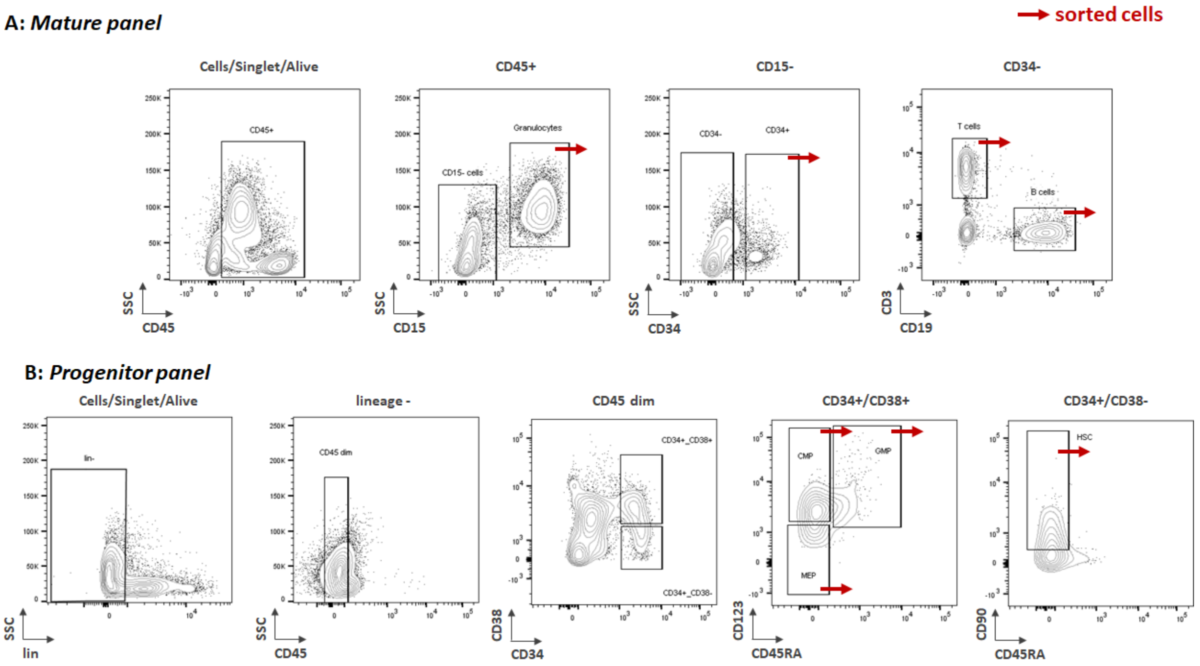

Supplemental Figure 1: Gating strategy for sorting BM subpopulations from individuals with CH carrying *ASXL1* mutations.

A: After dead cell exclusion, CD15$^+$ (granulocytes) cells were sorted, the negative fraction was then used to sort CD34$^+$ cells and the subsequent CD15$^-$CD34$^-$ fraction was used to sort CD3$^+$ (T-cells) and CD19$^+$ (B-cells). B: After dead cell exclusion, lineage negative cells (CD4$^-$CD8a$^-$CD15$^-$CD19$^-$CD235a$^-$) were selected and subsequently the CD45dim population. The CD34$^+$CD38$^+$ subpopulation was used to sort for CD45RA$^-$CD123$^+$ (CMPs), CD45RA$^+$CD123$^+$ (GMPs) and CD45$^-$CD123$^-$ (MEPs), while the CD34$^+$CD38$^-$ subpopulation was used to sort for CD45RA$^-$CD90$^+$ (HSCs).

Epigenetic modifier

Gene	CH, %	MDS, %	sAML, %
DNMT3A	57,5	28,3	25,2
TET2	29,1	42,4	21,1
ASXL1	8,7	22,8	26,8
IDH2	1,6	1,1	13,8
EZH2	0,0	5,4	4,9
IDH1	0,0	3,3	6,5
ASXL2	0,0	1,1	0,8
KDM6A	0,0	0,0	0,8

Splicing factor

Gene	CH, %	MDS, %	sAML, %
SRSF2	1,6	18,5	24,4
SF3B1	3,9	28,3	8,9
U2AF1	1,6	9,8	10,6
DDX41	0,0	4,3	2,4
ZRSR2	1,6	4,3	0,0
U2AF2	2,4	0,0	0,0

Cohesin Complex

Gene	CH, %	MDS, %	sAML, %
STAG2	0,0	6,5	21,1
RAD21	0,8	3,3	3,3

Signaling

Gene	CH, %	MDS, %	sAML, %
NRAS	0,0	2,2	22,8
FLT3	0,0	2,2	21,1
KRAS	0,8	3,3	10,6
PTPN11	0,0	0,0	13,0
JAK2	2,4	4,3	4,1
CBL	0,0	6,5	1,6
MPL	0,0	6,5	0,8
ETNK1	0,0	5,4	0,0
SH2B3	0,8	3,3	0,0
CSF3R	0,0	1,1	2,4
BRAF	0,0	2,2	0,8
KIT	0,0	1,1	1,6
MYD88	1,6	0,0	0,0
NOTCH1	0,8	1,1	0,0
STAT3	0,8	1,1	0,0
CXCR4	0,8	0,0	0,0
SRP72	0,8	0,0	0,0
CALR	0,0	1,1	0,0

Tumor Suppressor

Gene	CH, %	MDS, %	sAML, %
TP53	2,4	14,1	7,3
WT1	0,8	1,1	12,2
ETV6	0,0	0,0	4,9
PHF6	0,8	0,0	1,6

Other

Gene	CH, %	MDS, %	sAML, %
NPM1	0,0	0,0	19,5
PPM1D	5,5	0,0	0,0
SETBP1	0,0	4,3	0,8
TERT	0,8	1,1	0,0
PIGA	0,8	0,0	0,0
HNRNPK	0,0	0,0	0,8

Transcription factor

Gene	CH, %	MDS, %	sAML, %
RUNX1	1,6	19,6	31,7
BCOR	1,6	5,4	15,4
BCORL1	1,6	3,3	4,1
CEBPA	0,0	1,1	3,3
NFE2	2,4	0,0	0,0
ZBTB7A	1,6	1,1	0,0
GATA2	0,0	0,0	2,4
GATA1	0,0	0,0	0,8

Supplemental Figure 2: Frequency of mutations in individuals with CH, MDS or sAML, grouped according to functional category

Classification of genetic variants into functional categories was performed according to Döhner et al., N Engl J Med 2015; 373:1136–1152.

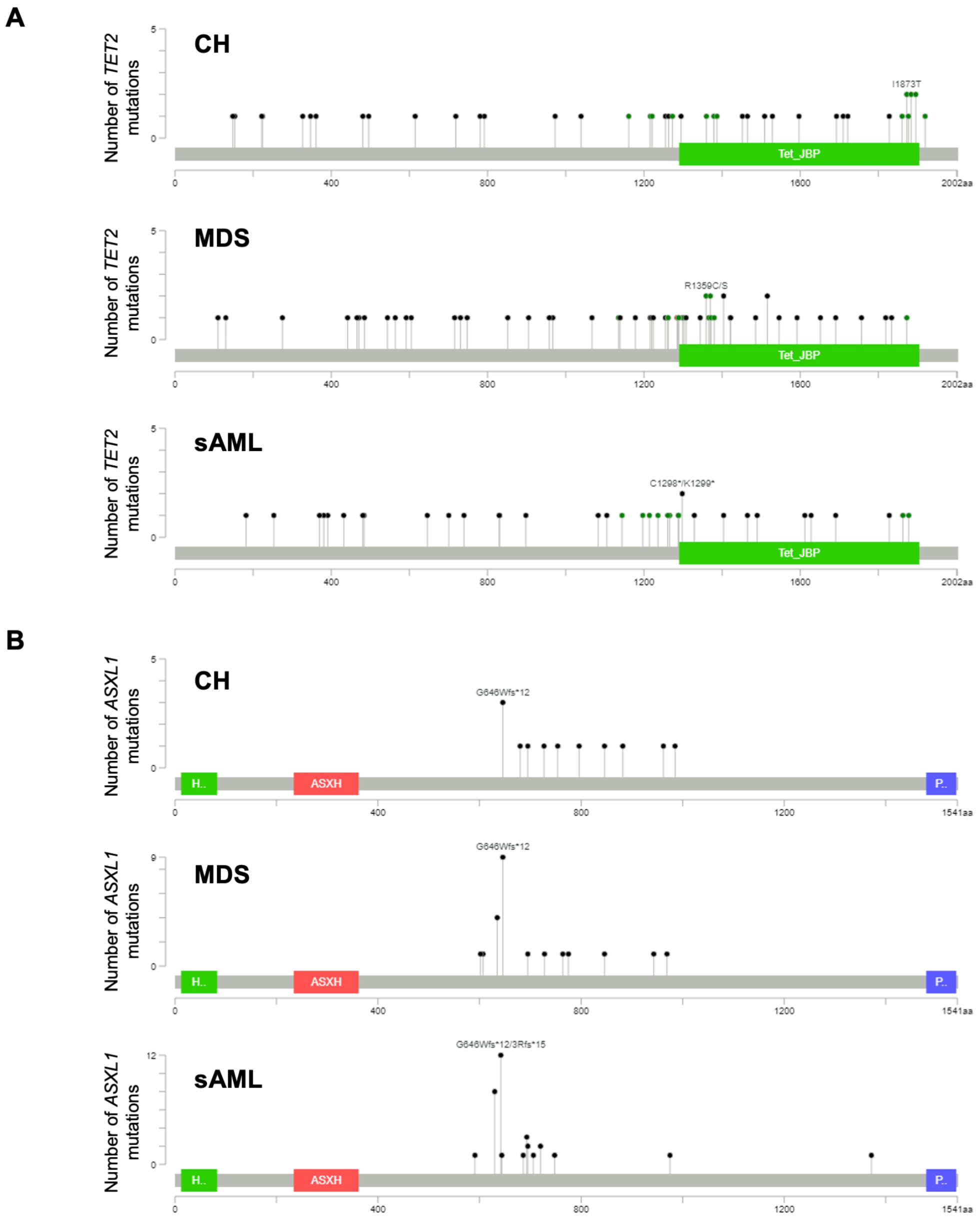

Supplemental Figure 3: Intra-genic localization of variants in CH, MDS and sAML.

(A) *TET2* variants; (B) *ASXL1* variants. Green dots indicate missense mutations, black dots indicate InDels, brown dots indicate nonsense mutations. Plots were generated using the MutationMapper tool (Gao et al., Sci. Signal. 2013 and Cerami et al., Cancer Discov. 2012).
